# The Antibacterial Type VII Secretion System of Bacillus subtilis: Structure and Interactions of the Pseudokinase YukC/EssB

**DOI:** 10.1128/mbio.00134-22

**Published:** 2022-09-26

**Authors:** Matteo Tassinari, Thierry Doan, Marco Bellinzoni, Maïalene Chabalier, Mathilde Ben-Assaya, Mariano Martinez, Quentin Gaday, Pedro M. Alzari, Eric Cascales, Rémi Fronzes, Francesca Gubellini

**Affiliations:** a Unité de Microbiologie Structurale, Institut Pasteur, UMR 3528, CNRS, Université de Paris, Paris, France; b Sorbonne Université, Collège Doctoral, Paris, France; c Laboratoire d’Ingénierie des Systèmes Macromoléculaires, Institut de Microbiologie, Bioénergies et Biotechnologie, UMR 7255, CNRS, Aix-Marseille Université, Marseille, France; d Institut Européen de Chimie et Biologie, Structure et Fonction des Nanomachines Bactériennes, Pessac, France; e UMR 5234, CNRS, Université de Bordeaux, Bordeaux, France; Newcastle University; Fred Hutchinson Cancer Center

**Keywords:** type VIIb secretion system, *Bacillus subtilis*, bacterial competition, pseudokinase, crystallographic structure, bacterial two-hybrid assay, interaction network, protein complexes, pseudokinases, secretion systems, structural biology

## Abstract

Type VIIb secretion systems (T7SSb) were recently proposed to mediate different aspects of *Firmicutes* physiology, including bacterial pathogenicity and competition. However, their architecture and mechanism of action remain largely obscure. Here, we present a detailed analysis of the T7SSb-mediated bacterial competition in Bacillus subtilis, using the effector YxiD as a model for the LXG secreted toxins. By systematically investigating protein-protein interactions, we reveal that the membrane subunit YukC contacts all T7SSb components, including the WXG100 substrate YukE and the LXG effector YxiD. YukC’s crystal structure shows unique features, suggesting an intrinsic flexibility that is required for T7SSb antibacterial activity. Overall, our results shed light on the role and molecular organization of the T7SSb and demonstrate the potential of B. subtilis as a model system for extensive structure-function studies of these secretion machineries.

## INTRODUCTION

The type VII secretion systems (T7SS) are multiprotein machines initially identified in the human pathogen Mycobacterium tuberculosis as responsible for the secretion of effectors and crucial for virulence (1–3). Further studies identified and characterized T7SS-like machines in several mycobacterial species (4–21) and in other actinobacteria (Nocardia, Corynebacteria, and Streptomyces) (22, 23), as well as in *Firmicutes* (24–33). Given their limited similarity, T7SS were then classified into two different families: type VIIa secretion systems (T7SSa) in *Actinobacteria* and T7SSb in *Firmicutes*. With the exceptions of an ATPase of the FtsK/SpoIIIE family with similar topology and domain organization and of a ubiquitin fold domain, T7SSa and T7SSb comprise distinct components: EccA, -B, -C, -D, and -E and MycP for T7SSa and EsaA (YueB), EsaB (YukD), EssA (YueC), EssB (YukC), and EssC (YukB) for T7SSb (*Bacillus* nomenclature in parentheses).

The recent structures of the mycobacterial ESX-3 and ESX-5 T7SSa revealed the overall architecture of the secretion apparatus and how the different subunits interact and started to shed light on the secretion mechanism of these machines (17, 19–21, 34). In contrast, little is known on the structure of the T7SSb. A putative model was proposed, based on the current knowledge of the S. aureus T7SSb and on extrapolations from T7SSa structures (35). The Staphylococcus aureus T7SSb ATPase EssC (*Sa*EssC) shares homology with its T7SSa counterpart in their central portions, including the transmembrane domain and four nucleotide binding sites (36). However, T7SSb ATPases possess two additional N-terminal forkhead-associated (FHA) domains of unknown function that are missing in T7SSa ATPases (37–39). The polytopic membrane subunit EsaA presents a large extracellular domain. The structures of the Streptococcus gallolyticus and S. aureus EsaA (*Sg*EsaA and *Sa*EsaA, respectively) extracellular domains demonstrate an elongated conformation, capable of spanning the peptidoglycan layer (40, 41). While *Sg*EsaA was proposed to control effector translocation through the cell wall, the tip structure of *Sa*EsaA suggested a role in cell-cell contacts. The structures of the S. aureus and Geobacillus thermodenitrificans EssB soluble domains revealed dimeric organization and a pseudokinase (PK)-like fold of the cytosolic domain (42, 43). Based on the presence of FHA and pseudokinase-like domains, an interaction between EssB and EssC was hypothesized (42). Although little is known on the organization of T7SSb components, EssB was shown to contact the EsaA subunit, and it copurified with EssA, EssC, EssD, EsaA, and EsxA in a dodecyl maltoside (DDM)-solubilized complex in S. aureus (44, 45).

At the physiological level, mycobacterial T7SSa have been shown to fulfill distinct functions, including the secretion of virulence factors, transfer of DNA, uptake of metal ions, and preservation of membrane integrity (16, 46–53). In *Firmicutes*, T7SSb were initially linked to persistence and virulence (25, 54, 55), and their antibacterial role was recently reported (56–59). Antibacterial effectors seem to be engaged in the T7SSb via different pathways (31, 56, 57, 59, 60). In S. aureus, several T7SSb substrates were identified, such as EsaD, EsxB, EsxC, EsxD, and TspA (35). EsaD, a DNase that is counteracted by EsaG, is recruited to the T7SSb by the EsaE chaperone. The EsaDE complex, as well as the effectors EsxB, EsxC, and EsxD, interacts with the T7SSb machine through the cytosolic domain of the EssC ATPase (56, 61). However, the modality of TspA recruitment by the T7SSb is still unknown. TspA is among the many LXG effectors that are secreted through the T7SSb in Gram-positive bacteria. The LXG proteins belong to the broad family of polymorphic toxins. Their N-terminal domains adapt the toxins to their delivery apparatus (62), while the C-terminal domains harbor their toxic activity. LXG genes usually have neighboring regions encoding cognate immunity proteins that avoid self-toxicity. In Streptococcus intermedius, WXG100-like proteins emerged as candidates for the recruitment of the LXG effectors on the secretion machine (57). However, their mode of recruitment and interaction with the T7SSb secretion machine remain largely unclear.

Six LXG proteins were identified in B. subtilis (60), including the YeeF DNase and the YobL, YxiD, and YqcG RNases, which cause growth inhibition when produced in Escherichia coli (63, 64). Recently, it was proposed that LXG effectors in B. subtilis mediate intraspecies competition, promoting spatial segregation in biofilms (65). Here, using as a model B. subtilis and its LXG effector YxiD, we characterize the molecular basis of T7SSb’s function in bacterial competition. We show that this LXG effector interacts with the YukC membrane protein, which has a pivotal role in the B. subtilis T7SSb. Finally, we report the crystallographic structure of the YukC dimer, highlighting a novel structural arrangement and its flexibility, corroborated by further *in vivo* analyses.

## RESULTS

### T7SSb-dependent bacterial competition in B. subtilis.

**FIG 1:**
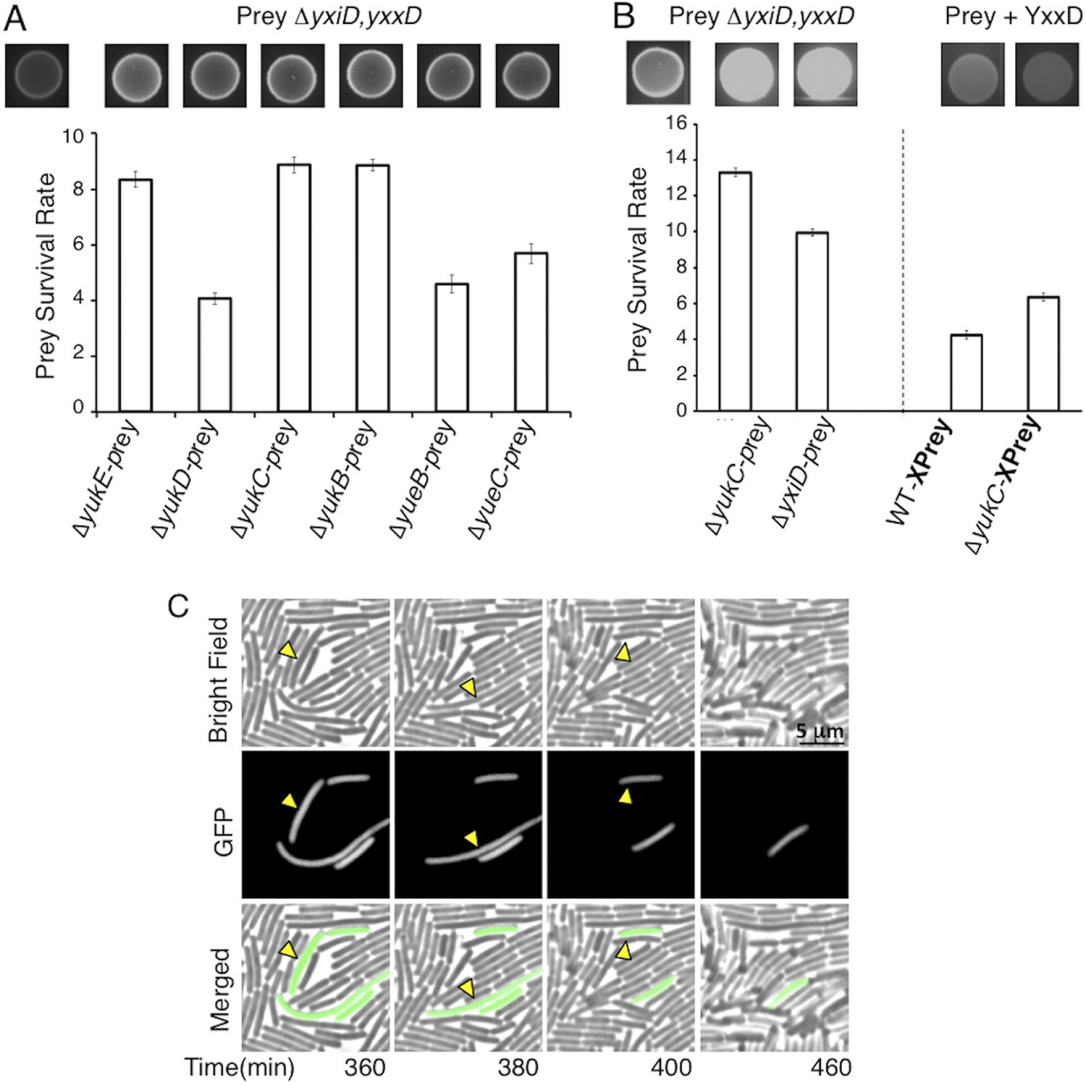
B. subtilis T7SSb-dependent competition. (A) Competition assay using the fluorescent strain B. subtilis Δ*yxiD-yxxD*::*gfp* as the recipient. The wild-type B. subtilis 168 CA strain and the indicated T7SSb mutant derivatives were used as attackers. Top, fluorescence of bacterial spots on agar plate after overnight competition. The spot on the far left resulted from the competition of the prey with the wild=type strain. The other spots were obtained by using attackers with the mutations indicated below. Bottom, prey survival rate plot, indicating that 4 to 9 times more cells survive when the *yuk* operon genes are deleted in the attacker. The values correspond to the competition spots shown above each plot. (B) Top, fluorescence of bacterial spots on agar plates after overnight competition using a standard prey (left) or a strain complemented for the immunity protein YxxD (right). As a control, the wild-type strain *vs* the standard prey was used (first spot on the left). Bottom left, prey survival rate plot, showing similar efficiencies when using the Δ*yxiC* attacker or one depleted of the YxiD toxin (10 to 12 times more cells than in the competing WT/Δ*yxiD-yxxD*::*gfp* strain). Bottom right, the YxxD-complemented prey survived the wild-type or the Δ*yxiC* attacker competition to similar degrees. (C) Effect of YxxD complementation in the recipient strain. In competition assays, the survival of the complemented prey having the *yxxD* immunity gene reinserted (strain B. subtilis Δ*yxxD-yxiD amyE*::*yxxD*) was similar for Δ*yukC* and wild-type attacker strains. (C) Frames from Movie S1 showing the disappearance of recipient cells from the bright field and fluorescence (GFP) images, as well as their composite images. Examples of cells disappearing in the next frame are highlighted with yellow arrowheads.

T7SSb are involved in bacterial competition in S. aureus and S. intermedius (56, 57, 60). To investigate whether the T7SSb confers antibacterial activity on B. subtilis, we focused on the LXG effector-immunity protein pair YxiD-YxxD (63, 66). YxiD shows cytotoxic activity when overproduced in E. coli and is neutralized when bound to its immunity protein YxxD (63). Hence, we engineered a B. subtilis recipient (prey) strain in which the endogenous *yxiD-yxxD* locus was replaced by an inducible green fluorescent protein (GFP)-encoding cassette, in order to visualize living cells by fluorescence. After overnight incubation in contact with wild-type B. subtilis, the fluorescence from the B. subtilis Δ*yxiD-yxxD* strain was very low (Fig. 1A, spot at top left), demonstrating that this strain was outcompeted by the wild type. In contrast, deletion of T7SSb genes in the attacker strain resulted in the survival of the recipient, as observed by fluorescent spots (Fig. 1A, top). The efficiency of competition was quantified by counting the surviving CFU on selective medium. The rate of prey survival was calculated as the number of survivors obtained with the T7SSb mutant versus the T7SSb wild-type (WT) attacker. Comparison with this value revealed that 4 to 9 times more cells survived the overnight coculture when the attacker strains had an impaired T7SSb than when the wild-type B. subtilis strain 168 was used (Fig. 1A, bottom). The lower efficiency of competition of these mutant strains was not due to any growth defect of the T7SSb mutants, as these cells exhibited growth kinetics comparable to that of the parental wild-type strain in nutrient-rich or nutrient-limiting medium (29). To exclude possible polar effects of these deletions, we tested the production of YueB, which is encoded by the fifth gene of the *yuk/yue* operon and serves as a receptor for phage SPP1 (67). We observed that the Δ*yukE*, Δ*yukD*, Δ*yukC*, and Δ*yukB* strains were lysed by SPP1, demonstrating that these upstream deletions did not affect the production of either YueB or other T7SSb subunits (Fig. S1 in the supplemental material). To further confirm that the competition was due to the transport of the YxiD effector into the target recipient cell, we used an attacker strain with this LXG effector deleted. In this case, comparable numbers of survivors were obtained when using Δ*yukC* and Δ*yxiD* attacker strains (Fig. 1B, left). Furthermore, complemented recipient cells producing the antitoxin YxxD (also called “PreyX”) survived overnight when cocultured with the wild type or with the T7SSb-impaired Δ*yukC* strain (Fig. 1B, right). Thus, we conclude that the cytotoxic effect of the attacker on the recipient depends on a functional T7SSb and that YxxD confers immunity to YxiD toxicity.

10.1128/mbio.00134-22.1 FIG S1 Competition assay controls. Effects of T7SSb gene deletions on the ability of SPP1 phage to lyse B. subtilis. Phage-induced bacterial lysis, mediated by the protein YueB, is observed in strains where the genes upstream from *yueB* in the T7SSb operon were each deleted (namely, B. subtilis Δ*yukE*, B. subtilis Δ*yukD*, B. subtilis Δ*yukC*, and B. subtilis Δ*yukB* mutants). Instead, *yueB* gene deletion causes resistance of B. subtilis to SPP1 lysis. Download FIG S1, JPG file, 0.04 MB. Copyright © 2022 Tassinari et al. 2022 Tassinari et al. https://creativecommons.org/licenses/by/4.0/ This content is distributed under the terms of the Creative Commons Attribution 4.0 International license.

10.1128/mbio.00134-22.9 MOVIE S1 Live bacterial competition between wild-type B. subtilis attacker and Δ*yxiD-yxxD* recipient cells. Competing bacteria were imaged every 20 min during overnight growth at 30°C on agar pads using phase-contrast and epifluorescence microscopy. The recipient strain B. subtilis Δ*yxiD-yxxD* shows GFP fluorescence, while the attacker (wild-type B. subtilis) has no fluorescence. Download Movie S1, AVI file, 3.7 MB. Copyright © 2022 Tassinari et al. 2022 Tassinari et al. https://creativecommons.org/licenses/by/4.0/ This content is distributed under the terms of the Creative Commons Attribution 4.0 International license.

To better document this competition, we recorded the interaction between B. subtilis attacker and recipient cells by fluorescence time-lapse microscopy (Fig. 1C, Movie S1). The images in Fig. 1C show sequential lysis of isolated recipient cells surrounded by wild-type attackers. At the 7-h point in Movie S1 (i.e., 8 h after the competing cultures were mixed), the fluorescent bacteria are confined into small patches. In contrast, the fluorescent recipients grew steadily without any noticeable killing event when incubated with Δ*yukC* attacker cells (Movie S2), confirming that B. subtilis 168 can outcompete same-species cells by secreting LXG effectors through a functional T7SSb.

10.1128/mbio.00134-22.10 MOVIE S2 Live-bacterial competition between B. subtilis Δ*yukC* attacker and Δ*yxiD-yxxD* recipient cells. Competing bacteria were imaged as described in the legend to Movie S1, using a Δ*yukC* strain of B. subtilis as the attacker. Download Movie S2, AVI file, 3.5 MB. Copyright © 2022 Tassinari et al. 2022 Tassinari et al. https://creativecommons.org/licenses/by/4.0/ This content is distributed under the terms of the Creative Commons Attribution 4.0 International license.

### The interaction network of B. subtilis T7SSb.

**FIG 2:**
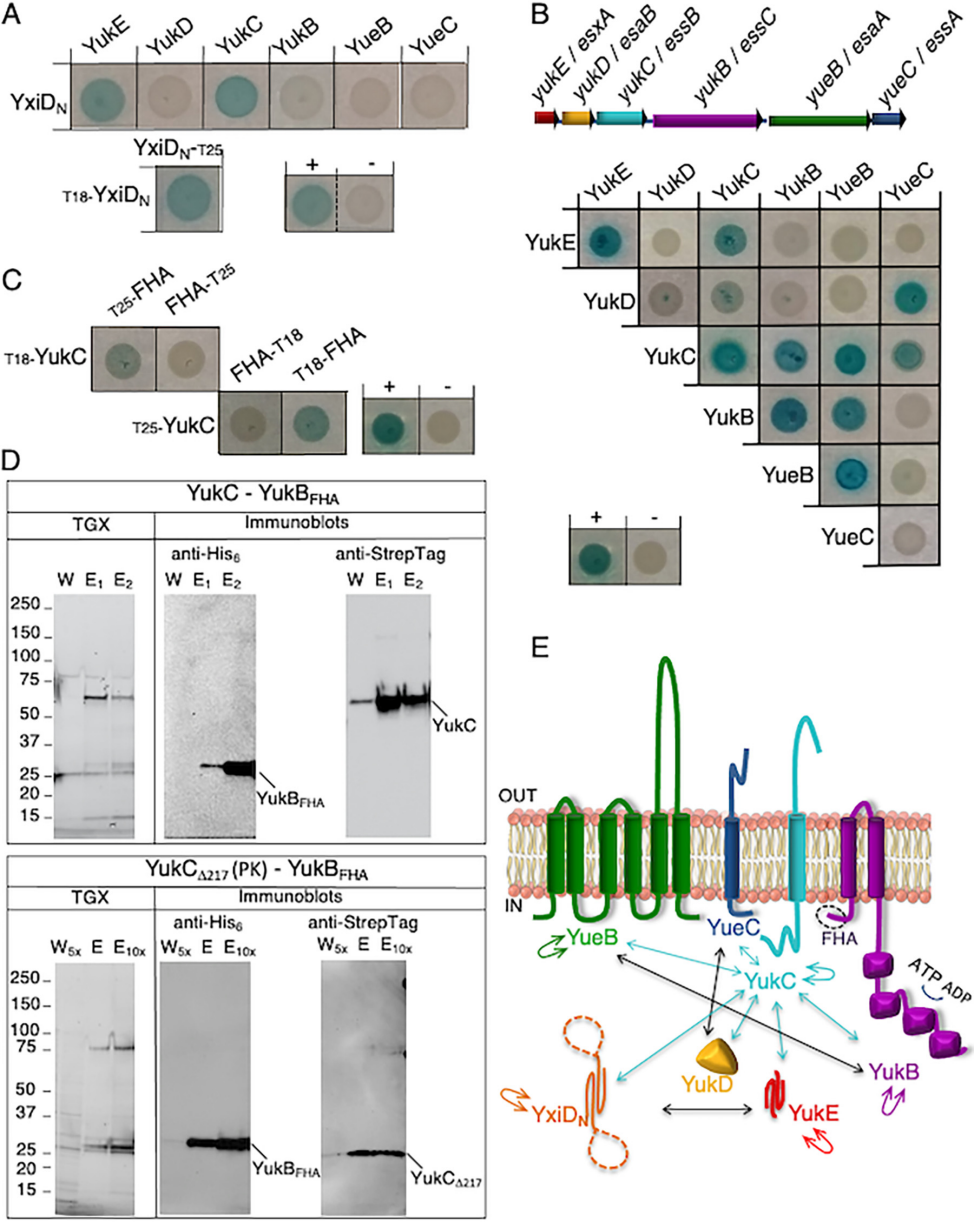
Binary protein interactions of T7SSb subunits and substrates. (A) Top, BACTH assay shows direct interaction of the YxiD N-terminal region (YxiD_N_) exclusively with YukE and YukC among the T7SSb subunits. Bottom, YxiD_N_ self-interaction (left) and the positive and negative controls (right). (B) Top, B. subtilis T7SSb (*Bs*T7SSb) operon gene organization. The gene nomenclatures for both B. subtilis (in bold) and S. aureus are shown for clarity. Bottom, a blue color of E. coli spots shows interaction between specific BACTH assay-tagged *Bs*T7SSb subunits. Representative positive and negative controls are shown. (C) BACTH assays showing YukC interaction with the N-terminal domain of YukB containing the two FHA regions with BACTH assay tags at the N or C terminus. (D) Copurification of YukC-YukB constructs. Top, the C-terminally Strep-tagged YukC coeluted with the YukB_FHA_ construct. The results of SDS-PAGE of wash (W) and elution (E_1_ and E_2_) samples from the second affinity column were revealed using the TGX stain-free method (left). Immunoblotting was performed on the same gel using antibodies recognizing the His_6_ tag and the Strep tag to detect YukB_FHA_ and YukC, respectively. Bottom, double affinity chromatography yielding copurification of the YukB_FHA_ construct with the pseudokinase domain (PK) of YukC (YukCΔ217 construct). In this case, the product of the wash step was concentrated 5 times (W_5x_) to better visualize the presence of proteins in this fraction. The elution fractions were pooled and loaded on the gel before and after concentrating 10 times (E and E_10x_). As before, TGX staining and immunoblots are shown, where anti-His_6_ and anti-StrepTag antibodies recognized YukB_FHA_ and YukCΔ217, respectively. (E) Schematic of binary interactions between the T7SSb components and with the YxiD_N_ construct. The T7SSb components are represented according to their predicted topology. YukC and its interactions are represented in cyan. Black arrows represent interactions between other T7SSb subunits.

We then investigated how LXG effectors interacted with the T7SSb complex. For this purpose, we used the bacterial two-hybrid (BACTH) assay (68). The target proteins were fused to the adenylate cyclase T18 or T25 fragments either at their N or C terminus, circumventing possible steric hindrance on a specific extremity of the proteins (Table S2). The toxic activity of the LXG effector YxiD is localized at the C terminus (63), while the N-terminal 97-amino-acid region (YxiD_N_) is predicted to be the trafficking domain (60) and to fold as an α-helical bundle. To avoid toxicity, we used the YxiD_N_ portion fused to the T18 or T25 fragments in BACTH assays. These constructs, tested against all the other T7SSb components, interacted exclusively with the WXG100 YukE substrate and the YukC membrane protein (Fig. 2A and E), suggesting that the pseudokinase participates in recruiting the LXG effectors. Also, we observed that YxiD_N_ self-interacts (Fig. 2A, bottom), similarly to the homologous LXG effector YeeF (64) and the WXG100 YukE substrate (28, 69).

10.1128/mbio.00134-22.7 TABLE S2 Plasmids used in this study. Download Table S2, PDF file, 0.2 MB. Copyright © 2022 Tassinari et al. 2022 Tassinari et al. https://creativecommons.org/licenses/by/4.0/ This content is distributed under the terms of the Creative Commons Attribution 4.0 International license.

To gain insight into the molecular organization of the secretion apparatus, we also investigated binary interactions between the B. subtilis T7SSb subunits by BACTH assays. All the target proteins interacted with at least one other subunit and/or with themselves, indicating that they were produced and properly folded in the E. coli strain used for the assay (Fig. 2B, Fig. S2A and B). We detected self-interactions for the YukC, YukB, and YueB subunits, as well as for the YukE substrate, in agreement with published data on homologous proteins (39, 42, 44, 69, 70). As expected from the predicted topology of YukC, only the constructs having the BACTH assay tags at the cytoplasmically located N terminus interacted (Fig. S2C). In contrast, no oligomerization was observed for YueC and YukD (Fig. 2B). Pairwise tests detected several novel interactions, including YukD-YueC and YukB-YueB. Noticeably, YukC appeared to interact with all the other proteins encoded on the *yuk* operon, with the YukC-YukD pair giving a weak but reproducible positive result (Fig. S2D). Interactions of YukC with the YueB and YukE subunits were further confirmed by copurification assays (Fig. S2E).

10.1128/mbio.00134-22.2 FIG S2 B. subtilis T7SSb interaction controls. (A) Table listing the combinations of T25 and T18 constructs giving positive interaction data. Those designated by an asterisk (*) gave the specific signal shown in Fig. 2B. (B) Images of the positive and negative controls are reported for each BACTH experiment showing protein interaction (Fig. 2B and Fig. S2A). The experiments are reported on the side of the corresponding control. (C) BACTH assay results on YukC oligomerization, confirming its predicted topology with the N terminus inside the cell and the C terminus outside. (D) Three different YukC-YukD interaction spots are reported, showing consistent positive interaction compared to the results for the controls. (E) YukC copurifications with YueB and YukE. Left, coexpressed YueB.strep and YukC.his were copurified by two subsequent affinity chromatography steps, first on a Strep-Tactin and then on a His-trap column. Results for Coomassie-stained SDS-PAGE of samples from the His-trap column purification are shown, including flowthrough (FT), wash (W), and elution (E) fractions. Immunoblotting of these fractions identifies the Strep-tagged YueB and His_6_-tagged YukC in the elution fractions (lanes E1 from E3 for the anti-Strep tag antibody), while fractions are pooled for the anti-His_6_ tag (E_pool_). Right, results for Coomassie-stained SDS-PAGE of samples from both His-trap and Strep-Tactin columns used to copurify YukC and YukE. Since the YukE His_6_ tag was not detected by immunoblotting, YukE’s identity was confirmed by mass spectrometry. For the Strep-Tactin column results: Ws, wash fraction. For the HisTrap column results: FTh, flowthrough fraction; Wh, wash fraction; E, elution fraction. Download FIG S2, JPG file, 0.5 MB. Copyright © 2022 Tassinari et al. 2022 Tassinari et al. https://creativecommons.org/licenses/by/4.0/ This content is distributed under the terms of the Creative Commons Attribution 4.0 International license.

Using various truncation mutants, we further showed that YukC interacts with the N-terminal region of the YukB ATPase, including two FHA domains (residues 1 to 256) (Fig. 2C). Interestingly, this region is conserved in the T7SSb homologs, while FHA domains are not found in T7SSa coupling proteins. The interaction between the YukB FHA N-terminal domain (construct YukB_FHA_) and YukC was confirmed by copurification (Fig. 2D, top). In addition, the YukB FHA region copurified with YukC’s pseudokinase (PK) domain, comprising 216 N-terminal residues (construct YukCΔ217) (Fig. 2D, bottom). Therefore, even though other domains may also interact, these data strongly suggest that the N-terminal YukB FHAs and YukC PK regions mediate the interaction between YukC and YukB. Taken together, these results provide a comprehensive picture of the network of T7SSb subunit interactions and identify YukC as their interaction hub (Fig. 2E).

### The crystallographic structure of YukC.

**FIG 3:**
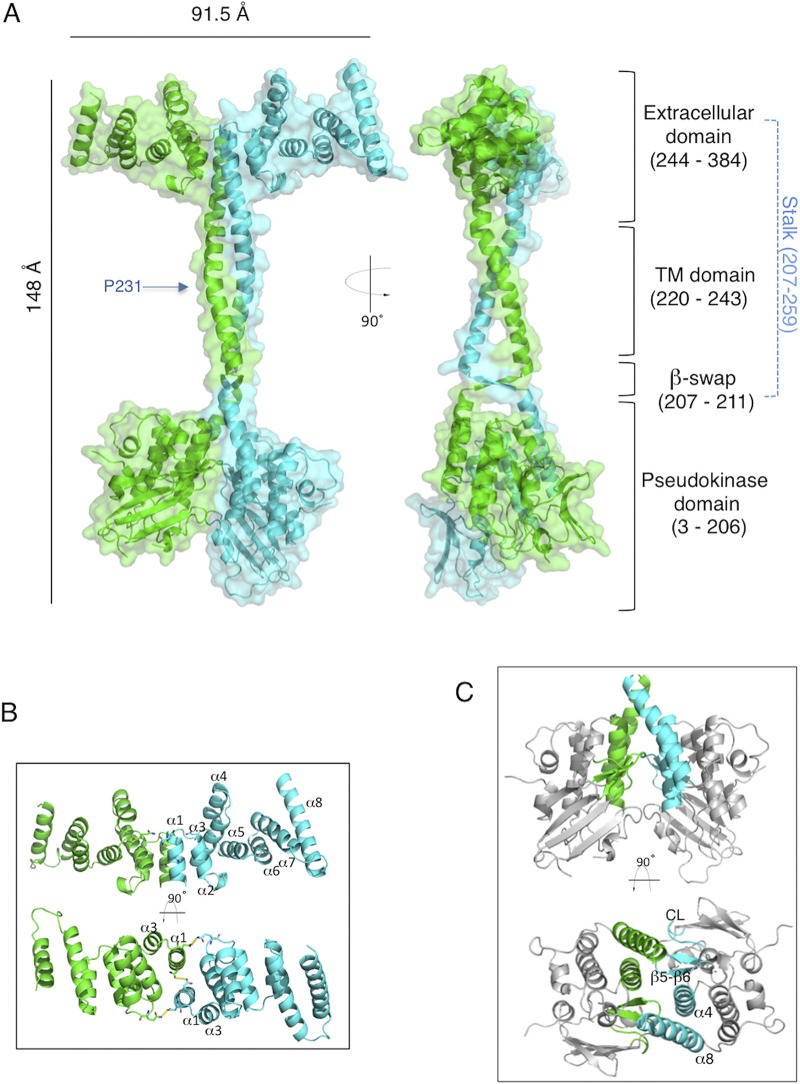
Structure of YukC. (A) Atomic model of YukC crystallographic structure (PDB ID 6Z0F). Left, front view. Right, side view. The YukC dimer is rendered in cyan (chain A, 384 residues) and green (chain B, 377 residues). Four major domains of the proteins are indicated on the right: the pseudokinase domain (residues 3 to 206), the *β*-swap domain (residues 207 to 211), the transmembrane (TM) region (residues 220 to 243), and the extracellular domain (residues 244 to 384). The central stalk is indicated in blue, going from the intracellular to the extracellular region of YukC (residues 207 to 259). The proline at position 231 is indicated at the center of the TM region. (B) Side and top views of the intermolecular interactions between YukC monomers in the extracellular region. The numbering of all the α-helices is indicated in the side view (top), while only the interacting helices are numbered in the top view (bottom). (C) The side and top views of YukC pseudokinase domains are reported, highlighting the interactions in the PK dimer. Regions involved in the intermolecular interactions are depicted in cyan (chain A) and green (chain B). In the top view (bottom), the interacting regions are numbered, including helices α4 and α8, β-strands β5 and β6, and the connecting loop (CL).

YukC being a central component of the B. subtilis T7SSb, we sought to obtain structural information for it. Both soluble regions of the G. thermodenitrificans and S. aureus YukC homologs were previously crystallized, revealing a pseudokinase-like domain in the cytosol (42, 43). However, the lack of the central transmembrane domain in these structures left uncertainties on the function and structural organization of the T7SSb pseudokinases, limiting structure/function interpretation. We therefore purified the full-length YukC and subjected the protein to crystallization trials. Unfortunately, YukC resisted all attempts at crystallization. We reckoned that the last 38 residues at the C terminus, mostly positively charged, could interfere with crystallization. We therefore produced a YukC_413_ construct lacking these residues, which promptly crystallized. Its structure was solved by molecular replacement on a 2.6-Å-resolution data set (PDB identifier [ID] 6Z0F) (Fig. 3A, Table 1) using the coordinates of the soluble domains from G. thermodenitrificans EssB as search models (PDB IDs 2YNQ and 4ANO). In the crystal, YukC organized as a pseudosymmetrical dimer with chains A and B revolving around a virtual central axis (384 and 377 residues assigned, respectively). The final stretch of residues could not be traced due to the lack of supporting electron density (Fig. S3A). In the crystal packing, two YukC dimers were associated in an antiparallel arrangement, with the N-terminal region of one dimer contacting the C terminus of the other (Fig. S3B) in a nonphysiological organization.

**TABLE 1:** X-ray crystallographic data and refinement statistics for YukC_413_

Parameter^*a*^	Value(s) for YukC_413_^*b*^
Space group	C_2_
Unit cell parameters	
a, b, c (Å)	149.02, 83.41, 106.45
*α*, *β*, *γ* (°)	90, 108.16, 90
Resolution range (Å)	29.42–2.55 (2.86–2.55)
Wavelength (Å)	0.9677
No. of:	
Measured reflections	161,374 (9,835)
Unique reflections	26,268 (1,314)
Multiplicity	6.1 (7.5)
Completeness (%)	92.3 (62.1)
Avg I/σ〈I〉	4.7 (1.2)
*R*_merge_	0.227 (1.482)
CC_1/2_	0.979 (0.588)
Refinement statistics	
PDB ID	6Z0F
*R*_work_	0.235
*R*_free_	0.269
No. of non-H atoms in:	
Macromolecule	5,827
Water molecules	8
Avg no. of *B* factors	88.5
RMSD^*c*^	
Bond length (Å)	0.010
Bond angle (°)	1.27
MolProbity statistics^*c*^	
Clashscore	3.65
Ramachandran plot (%)	
Outliers	0.00
Favored regions	95.45
Rotamer	
outliers	3.30
C^β^ deviations	0.00

a Resolution limits were determined by applying an anisotropic cutoff via STARANISO, part of the autoPROC data processing software (98). CC1/2 is the Pearson's correlation coefficient. $R_{\text{merge}}=\;\Sigma_{h}|I_{h}-\langle I\rangle |/\Sigma_{h}I_{h}$, where *I_h_* is the intensity of reflection *h* and 〈I〉 is the mean intensity of all symmetry-related reflections. $R_{\text{work}}=\;\Sigma \left|\left|F_{o}\right|-\left|F_{c}\right|\left|/\Sigma \right|F_{o}\right|$, where *F_o_* and *F_c_* are the observed and calculated structure factor amplitudes. Five percent of the reflections were reserved for the calculation of *R*_free_.

b The data in parentheses refer to the highest-resolution shell.

c Calculated with MolProbity (100) within the Phenix crystallographic software suite.

10.1128/mbio.00134-22.3 FIG S3 Analysis of the YukC model. (A) The sequence of YukC is reported, with the residues in black corresponding to the ones present in the YukC_413_ construct (plus the Strep II tag, in red). The light-grey region was not included in the crystallized construct. The underlined residues are the ones assigned to the model (double underline, visible in both chains; single underline, visible only in chain A). (B) Head-to-tail arrangement of YukC dimers in the crystal packing. (C) Atomic model of YukC superimposed with the hydrophobic surface as calculated by using Chimera software (red, hydrophobic; blue, hydrophilic). (D) Top, structural alignment of the extracellular domains of YukC (cyan) and EssB from G. thermodenitrificans (PDB ID 2YNQ) (pink). Bottom, table illustrating the structure similarities of the proteins. (E) Analysis of YukC-YueB interaction. Left, scheme of YueB_INT_, where the large extracellular loop (dotted lines) is mutated into a short loop (solid line). Right, BACTH assay of the interaction between YukC and YueB_INT_. The blue spots show interaction between YueB_INT_ and YukC. Also, YueB_INT_’s interaction with the full-length YueB is shown. Positive and negative controls are reported. (F) Structural comparison between the pseudokinase domains of YukC (chains A, cyan) and the structures of the same region from S. aureus EssB (PDB ID 4ANN) (orange) and from G. thermodenitrificans (PDB ID 4ANO) (pink). Below, table illustrating their structure similarities. (G) Numbering of secondary structures in the YukC pseudokinase region. Download FIG S3, JPG file, 0.7 MB. Copyright © 2022 Tassinari et al. 2022 Tassinari et al. https://creativecommons.org/licenses/by/4.0/ This content is distributed under the terms of the Creative Commons Attribution 4.0 International license.

The YukC structure can be subdivided into four main regions from the N to the C terminus: the pseudokinase domain (residues 3 to 206), the *β*-swap domain (residues 207 to 211), the transmembrane (TM) domain (residues 220 to 243), and the extracellular domain (residues 244 to 384) (Fig. 3A). The symmetry of the structure is broken at the level of the pseudokinase domains, which are oriented differently in the two monomers. The TM region was defined based on the hydrophobicity profile (Fig. S3C), in accordance with the TMHMM algorithm prediction, and presents a cavity on its intracellular side. A central “stalk” crosses the different domains, starting from the β-swap and ending in the extracellular region (residues 207 to 259). The α-helices forming the stalk change their orientation inside the membrane (at the proline 231) and near the extracellular side (residues 241 to 242), where they form the top of the stalk.

### The extracellular domain.

The YukC extracellular domain presents an all-α-helical organization, the structure of which is not reminiscent of other proteins or domains of known function (DALI analysis at http://ekhidna2.biocenter.helsinki.fi/dali/). Overall, this region appears highly similar to that of G. thermodenitrificans EssB (*Gt*EssB; root mean square deviation [RMSD] = 1.5) (Fig. S3D) (42). It is composed of 8 α-helices interacting in coiled-coil motifs and extending approximately 40 Å from the membrane plane. In the extracellular region, the YukC monomers interact at the level of α-helix 1 and of the loop between helices α4 and α5 (Fig. 3B), contributing to YukC dimer stabilization. We tested whether this region might be involved in the interaction with YueB, which is the only T7SSb subunit having a large extracellular domain. Our bacterial two-hybrid assay results show that YukC still interacts with a variant of YueB that is missing 90% of its extracellular domain (YueB_INT_, lacking residues 31 to 817) (Fig. S3E). Since the other loops of YueB are relatively short, this result suggests that YukC and YueB may interact primarily through their TM regions, as proposed for the S. aureus homologs (45).

### The pseudokinase domain.

Structural comparisons between the PK domains of YukC and the homologous regions of EssB proteins from S. aureus (PDB ID 4ANN) and G. thermodenitrificans (PDB ID 4ANO) indicated a closer similarity to the latter (Fig. S3F), reflecting their levels of sequence identity (15% with S. aureus and 43% with G. thermodenitrificans). Nevertheless, the PK domains (described in detail in reference 42) exhibit an overall high similarity in the three models (Fig. S3F). Adding to the information given by the crystals of the monomeric PK from EssB, the YukC structure reveals that the PK dimers interact through four regions: α-helices α4 and α8 (K^83^-Q^99^ and Y^186^-T^206^), the linker between β-strands β5 and β6 (I^114^-F^124^), and the connecting loop (CL; P^68^-A^74^) (Fig. 3C, Fig. S3G). In spite of these contacts, the two PK domains do not contribute to the stability of the dimeric structure (see below).

To investigate whether the PK regions were able to bind and hydrolyze ATP, we compared the YukC sequence and structure to those of its T7SSb homologs (S. aureus and G. thermodenitrificans EssB proteins) and to other kinases or pseudokinases (identified as best matches on DALI [71]). The most important motifs characterizing Hanks-type kinases are partially missing in YukC pseudokinases (Fig. S4A). These include the glycine-rich P-loop coordinating the ATP β- and γ-phosphate, the VAIK motif, and the catalytic, Mg-binding, and activation loops (72). The structural comparison of YukC with PknB in complex with ATP (PDB ID 1O6Y) (RMSD = 4.2 Å for their pseudokinase domains) shows that YukC has an additional α-helix in the ATP-binding pocket, with a large side chain (F^26^) partially occluding the ATP binding site (Fig. S4B). In agreement with this, microscale thermophoresis assays show that YukC cannot bind ATP *in vitro*, in contrast to PknB (Fig. S4C). Taken together, these sequence and structural analyses, as well as *in vitro* ATP-binding assays, suggest that YukC is unable to bind ATP (and to hydrolyze it).

10.1128/mbio.00134-22.4 FIG S4 Pseudokinase domains in the YukC dimer. (A) Sequence alignment of B. subtilis YukC, its S. aureus and G. thermodenitrificans EssB homologs, and known kinases and pseudokinases identified based on best matches using a DALI search. These include kinases Sirk1, TNNI3K, Pim1, Bsk8, and Mycobacterium tuberculosis PknB (*Mt*PknB) and the pseudokinase M. tuberculosis MviN (*Mt*MviN). The motifs characterizing the Hanks-type kinases are highlighted: the P-loop, the VAIK motif with the conserved Lys, the conserved Glu, the catalytic loop, the Mg^2+^-binding region, and the activation loop. (B) Structural comparison between YukC and *Mt*PknB in complex with ATP (PDB ID 1O6Y). The degenerated N lobe presents an additional α-helix in the ATP-binding pocket of YukC, with the large side chain of F^26^ occluding the ATP-binding pocket. (C) Nanoscale differential scanning fluorimetry (nanoDSF) measurements comparing YukC (left) and the kinase PknB from *Mt*PknB (right), in the presence and absence of ATP/Mg^2+^. The fluorescence signal, expressed as the 350-nm/330-nm emission ratio, is plotted as a function of the temperature. The average signal from three replicates is reported (two for PknB with ATP). The difference in the melting temperature (*T_m_*) of each protein in the presence and in the absence of ATP is indicated at the bottom of the respective plots. (D) Alignment of chains A (cyan) and B (green). Their divergence—caused by the different orientations of the helices α8 in the PK domains—is indicated by a red asterisk. (E) Link between the asymmetrical K^205^-A^80^ interaction and the lifting up towards the membrane plane of one PK monomer compared to the other. The K^205^ residues are depicted in magenta. Download FIG S4, JPG file, 0.6 MB. Copyright © 2022 Tassinari et al. 2022 Tassinari et al. https://creativecommons.org/licenses/by/4.0/ This content is distributed under the terms of the Creative Commons Attribution 4.0 International license.

### The stalk and β-swap domains.

**FIG 4:**
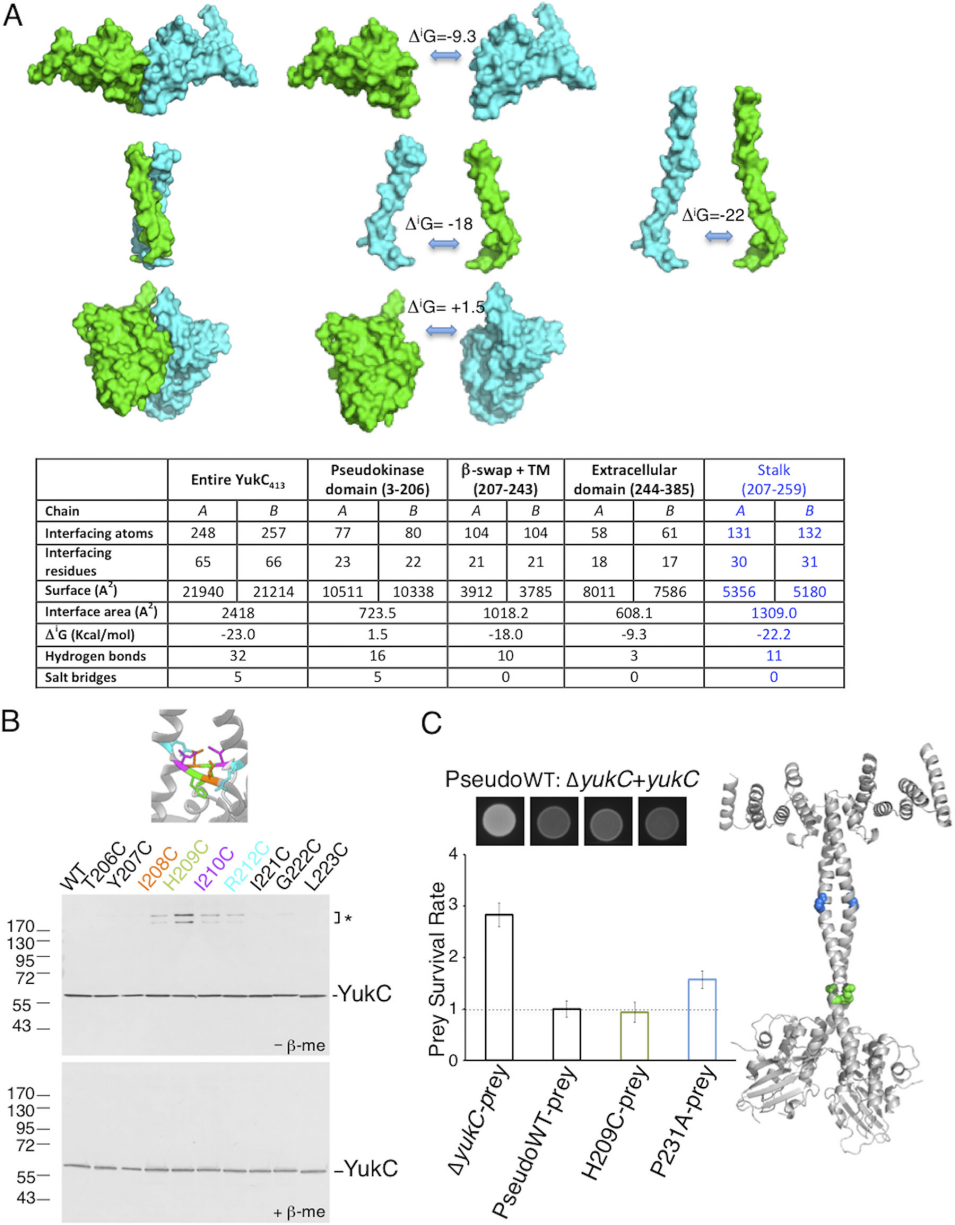
Structural bases of YukC dimer interactions. (A) Energetics of YukC monomer interactions. Chain A is shown in cyan, and chain B in green. Δ*^i^G* indicates the solvation free energy gain upon formation of the interface. Top, schematic of the different contributions to YukC dimer stabilization. Each YukC domain is shown separately (left and center) and their interactions compared to the stalk, including the β-swap, the TM region, and a portion of the extracellular domain (right). Bottom, results from dimer interface analysis by PISA. (B) *In vivo* validation of the YukC structure by disulfide cross-linking. Total extracts of B. subtilis cells producing the wild-type His_6_-YukC protein or the indicated cysteine variants were subjected to SDS-PAGE and immunodetection using anti-His antibody. Samples were treated (bottom gel, −β-me) or not (top gel, +β-me) with β-mercaptoethanol as a reducing agent. The asterisk and bracket on the right indicate bands corresponding to a disulfide bond YukC oligomer. The dimerizing residues are indicated with the same color code used in the scheme highlighting them in the YukC structure (top). (C) Left, effects of YukC mutations on bacterial competition. The fluorescent spots at the top were imaged after overnight competition. The corresponding values for the competition reactions are indicated below in the prey survival rate plot. The YukC-H209C mutation yields the same number of survivors as the Δ*yukC*+*yukC* complemented strain (PseudoWT), while a higher survival rate is observed when using the YukC-P231A strain as the attacker. Right, the YukC model is depicted with the positions of the mutated residues; H209 is in green and P231 in cyan. The same color code is used in the plot of the results of reactions with the corresponding mutants.

The YukC structure reveals two unexpected features: a β-swap domain and a twisting stalk. Analysis of the dimer’s interfaces on the PDBe PISA web tool shows that YukC dimerization is mainly governed by the intermolecular interactions occurring within the stalk (solvation free energy gain upon formation of the interface [Δ*^i^G*] = −22), including the TM domain (Fig. 4A). Despite the comparable interaction surfaces of the extracellular and the PK domains, their Δ*^i^G* values are significantly different, indicating that only the first one effectively contributes to the stability of the YukC dimer (Fig. 4A).

The *β-*swap is located below the membrane plane, where the monomers cross as antiparallel β-strands. The region that includes the *β-*swap domain has no structural homologue, as indicated by a DALI search (71). A hydrophobic patch between the *β-*swap and the transmembrane region is formed by three amino acids of each β-strand (I^208^, I^210^, and P^211^) and the W^214^ residues from the stalks (Fig. S5A). On both sides of the *β-*swap domain, the conserved residues K^205^, R^212^, and K^213^ define a positively charged region that may be involved in the protein’s interaction with the negatively charged polar heads of the membrane’s lipids (Fig. S5B).

10.1128/mbio.00134-22.5 FIG S5 Analysis of the stalk and β-swap in the YukC dimer. (A) Hydrophobic residues in the YukC structure calculated by the PyMol software are depicted in red. In the inset, the hydrophobic patch over the β-swap is highlighted. The H^209^ residue is rendered in blue. (B) Electrostatic potential surface of the atomic model of YukC, according to the coulombic law (violet, −10 kcal/mole; cyan, 0 kcal/mole; yellow, +10 kcal/mole). In the inset, the positive charges localized in the proximity of the β-swap are highlighted and the I^208^, H^209^, and I^210^ residues are shown in orange. (C) Above, the proline residues P211, P231, and P245 are depicted as cyan spheres to highlight their localization along the YukC stalk. The membrane region is shown in yellow. Below, the sequence logo shows the conservation of the YukC region, including the TM domain, based on the HMMER sequence alignment. The conservation of the three proline residues is highlighted. (D) The P231A mutation does not decrease YukC stability. YukC wild type (YukC.His) and its YukC.His^P231A^ mutant were expressed in E. coli, together with the membrane protein TseB as a control. Cell samples collected at different time points after protein synthesis was blocked by chloramphenicol were analyzed by Western blotting using antibodies directed against the His tag (YukC) and TseB (upper panels). The signals from YukC.His and YukC.His^P231A^, normalized by the signals from TseB, show similar decay rates for the two protein constructs (bottom). Download FIG S5, JPG file, 0.5 MB. Copyright © 2022 Tassinari et al. 2022 Tassinari et al. https://creativecommons.org/licenses/by/4.0/ This content is distributed under the terms of the Creative Commons Attribution 4.0 International license.

The *in vivo* structure of the *β-*swap was probed by cysteine-scanning analyses: residues 206 to 212 and 221 to 223 were individually replaced with cysteines, and the formation of disulfide bonds between the two monomers was assessed by denaturing SDS-PAGE under reducing and nonreducing conditions. Intermolecular disulfide bridges were detected in the I208C (a change of I to C at position 208), H209C, I210C, and R212C variants (Fig. 4B). The strongest disulfide bond formation was observed with the H209C substitution. With H^209^ being located in the middle of the *β-*swap, their side chains face each other (Cα-Cα distance in the YukC model = 5 Å). The proteins with I208C and I210C substitutions (Cα-Cα distance = 8.1 and 8.4 Å, respectively) presented weaker interactions. Surprisingly, the R212C variant was also able to form disulfide bonds in spite of the distance between these two residues in the crystallographic model (15.6 Å), suggesting that the YukC dimer may undergo a significant structural change in this area.

To test whether the H209C substitution in the swap region would affect T7SSb activity *in vivo*, we reintroduced either a wild-type copy of *yukC* or the *yukC* H209C allele onto the chromosome of a Δ*yukC* mutant (see Materials and Methods). In the first case, a YukC complemented strain was obtained (PseudoWT) (Fig. 4C). Although the complemented T7SSb of the PseudoWT was less functional than the wild-type one, it recovered partial activity, as indicated by a smaller number of competition survivors than for the Δ*yukC* strain (Fig. 4C). Interestingly, a similar prey survival rate was observed for the strain carrying the *yukC* H209C mutation. Using the same assay, we also tested the role of P^231^ in T7SSb functionality. Prolines are known to introduce flexibility into α-helices, potentially influencing signal transduction (73–76). Besides this, the P^231^ residue contributes to the major kink of the YukC’s TM region (Fig. 3A). The strain harboring the *yukC* P231A mutation yielded more survivors than the pseudo-wild-type strain (Fig. 4C). Altogether, these data show that the effector’s secretion through the T7SSb is affected by the mutation of the central transmembrane residue P^231^, potentially involved in signal transduction.

Interestingly, in the YukC structure, the orientation of the monomers diverges below the *β-*swap. This asymmetry is related to the different orientations of helix α8 in the two PK domains, favoring the H-bond between residues K^205^ of chain A and A^80^ of chain B (Fig. S4D, red asterisk). As a consequence, the PK domain of one monomer (chain B) is closer to the membrane plane than the other (Fig. S4E). This further supports structural flexibility in the region of YukC surrounding the *β*-swap. In the future, it will be interesting to investigate the physiological effects of mutating these residues.

## DISCUSSION

In this study, we investigate the T7SSb-driven bacterial competition in B. subtilis through the secretion of the LXG antibacterial effector YxiD. We identify two T7SSb components that directly interact with the YxiD effector and delineate an interaction network between T7SSb subunits, which puts forward a central role for the membrane pseudokinase YukC/EssB. The crystal structure of YukC highlighted new, unprecedented features, such as a stalk region that may provide YukC with intrinsic flexibility. Our analysis endorses B. subtilis as an ideal system for investigating functional and structural aspects of T7SSb by establishing that its organization revolves around YukC.

### T7SSb-dependent bacterial killing in B. subtilis.

Bacteria use distinct mechanisms to compete for conquering an ecological niche. In B. subtilis, contact-independent competition can occur through the production of specialized metabolites, such as bacillaene (77) and the lipopeptides plipastatin (78) and surfactin (79). Surfactin is required for biofilm formation and motility, but it also inhibits other bacteria, favoring the fitness of B. subtilis in some environments (80, 81). In addition, B. subtilis secretes toxins of the YD repeat protein family to inhibit the growth of other B. subtilis cells in a contact-dependent manner (82). Recently, LXG effectors were identified in B. subtilis, as in other *Firmicutes* (60, 83). They exhibit RNase activity when produced in E. coli (63) and play a role in bacterial biofilm organization (65). Using a B. subtilis strain lacking protection against the LXG effector YxiD as the recipient (B. subtilis Δ*yxiD-yxxD*), we showed that B. subtilis causes growth inhibition in a T7SSb-dependent manner (Fig. 1). Accordingly, the deletion of *yxiD* in the attacker or the production of its cognate antitoxin YxxD in the recipient yielded increased survival rates of the recipient cells after competition (Fig. 1B). The possibility to use a rapid B. subtilis functional competition assay opens new opportunities for thoroughly characterizing T7SSb-based antibacterial mechanisms. Notably, we show that the domesticated strain B. subtilis 168 produces a functional T7SSb. The discrepancy with what was previously proposed (28) may be explained by the difference in the sensitivities of the methods used to test T7SSb activity (detection of YukE in the extracellular milieu versus bacterial competition activity).

Although further studies are required to better understand the T7SSb-mediated antibacterial mechanism, time-lapse microscopy indicated that recipient cells lyse after contacting attackers, eventually surviving within small patches (Movie S1). In Movie S2, the prey and the Δ*yukC* strain appear to be growing at similar rates despite the absence of a functional T7SS in the latter, due to the presence of all of the LXG immunity proteins. It will be interesting to investigate further whether the presence of a functional T7SSb could represent an advantage for the prey over the Δ*yukC* strain under the conditions used in competition assays. We support, like Kobayashi (65), the hypothesis that B. subtilis T7SSb-mediated competition occurs in a contact-dependent manner.

### A T7SSb protein interaction network.

Our competition assay also suggests that *yukE* is required for efficient T7SSb-based killing in B. subtilis. This is in agreement with the recent proposal, based on the structure of M. smegmatis’s ESX-3, that substrate interactions may favor a secretion-competent state (10, 20). Furthermore, specific WXG100-like proteins were proposed to deliver the LXG toxins to the T7SSb machine in S. intermedius (57), reminiscent of the chaperone role of the EspG proteins in M. tuberculosis’s T7SSa (84, 85). Accordingly, we demonstrate that YukE interacts with the trafficking domain of the LXG effector YxiD, which also interacts with YukC (Fig. 1A). Therefore, a possible pathway for LXG effectors would include their selection by the WXG100(-like) proteins and their delivery to the T7SSb apparatus via contacts with the pseudokinase subunit.

Our BACTH and copurification analyses provide an important survey of protein-protein interactions within this secretion system, shedding light on fundamental aspects of T7SSb organization (Fig. 2B and E, Fig. S2). Importantly, key YukC interactions with YukE, YukB, and the multispanning membrane protein YueB were confirmed by copurification (Fig. S2D). Additional interactions include complex formation between the YueB polytopic membrane protein and the YukB ATPase and between the YueC membrane protein and the YukD ubiquitin-like cytosolic subunit. A ubiquitin-like component is also present in the T7SSa, as a domain of the multispanning protein (EccD in the ESX systems). These regions may play a similar role in the T7SS, albeit the ubiquitin-like domain is associated with the cytoplasmic domain of the ATPase EccC in the ESX-3 model (19, 20), while YukD did not show direct interaction with the YukB ATPase by double-hybrid assay.

YukC also interacts with the YukB ATPase and with the YueB polytopic protein, reminiscent of the role of EccB3 in ESX-3 (19, 20). While YueB and YukC appear to interact via their transmembrane regions, the YukB-YukC interaction involves their cytoplasmic domains (YukB FHA and YukC PK) (Fig. 2C and D). FHA domains usually recognize and bind phospho-threonine residues on several eukaryotic and prokaryotic proteins (38, 86). However, the phosphorylation of the threonine residues on YukC’s domain requires a eukaryotic-like Ser/Thr kinase (eSTK) (or Hanks-type kinase), such as PrkC in B. subtilis (87). Since the YukC-YukB_FHA_ interaction was tested in E. coli, in which, to our knowledge, no typical eSTK is present (88), this suggests that the FHA-PK interaction in T7SSb is phosphorylation independent. Indeed, residues responsible for the binding of phosphorylated targets are not conserved in either the FHA of YukB (38) or those of EssC from S. aureus and G. thermodenitrificans (PDB IDs 1WV3 and 5FWH, respectively) (37, 39).

### YukC’s structure and its functional implications.

The C-terminal portion of YukC extends 40 Å above the membrane plane and participates in stabilizing the YukC dimer. Since this region does not seem to be involved in the interactions with other subunits, it may potentially help the positioning of the T7SSb machine through interactions with the peptidoglycan, as proposed for the S. aureus homolog EssB (42). The AlphaFold2 prediction proposes that the C terminus of YukC that is missing in our model would form a straight, 50-nm-long α-helix rich in positively charged residues (89), suggesting that this region might cross the peptidoglycan, reaching the bacterial surface.

Despite the asymmetric contacts observed in the YukC crystal packing (Fig. S3B), the YukC monomers show overlapping structures (within the experimental error). This can be ascribed to conformational stability of this domain when in contact with other proteins. In the PK domain of monomer A, the bending of helix α8 favors the asymmetric K^205^-A^80^ interaction and the uplifting of one PK domain compared to the other (Fig. S4D and E). This hinge region would allow the PK domains to alternate in “up” or “down” positions through rigid-body movement (Fig. S4E). Moreover, since the PK domains interact directly with the YukC ATPase (Fig. 2), we speculate that their motion may be involved in coupling ATPase conformational changes to other secretion events in the T7SSb.

The low Δ*^i^G* value of the PK dimer interface suggests that the dimer-monomer transition could be easily modulated by interacting T7SSb subunits or substrates. The YukC intracellular motion is further supported by our cysteine-scanning analysis. Indeed, whereas in the crystallographic model, R^212^ residues are located relatively far from each other (15.6 Å), the R212C variant forms disulfide bridges *in vivo* (Fig. 4B). Therefore, the *β*-swap may have enough lateral flexibility to allow this disulfide bridge to form during YukC activity. The hydrophobic patch located between the stalk and the TM region (residues I^208^, I^210^, P^211^, and W^215^) (Fig. S5A) could play a role in this mechanism, especially in the case of T7SSb assembly in lipid rafts, as proposed in Mielich-Suss et al. (90). Similarly to T7SSa (10), ATP hydrolysis was proposed to involve conformational changes in the cytoplasmic domains of the T7SSb ATPase (36). YukC, with its intracellular dynamics, is ideally placed to link the sites of these ATP-driven events to the rest of the complex.

Besides revealing key roles of YukC in T7SSb secretion, the structure of the stalk in YukC represents, to our knowledge, the very first visualization of the transmembrane region of a membrane histidine (pseudo)-kinase. Despite their functional differences in the catalytic domain, these kinases and pseudokinases have mostly been found to be involved in signal transduction. Indeed, it was originally proposed that S. aureus EssB could mediate signal transduction (42). In a recent model of DesK, a well characterized histidine kinase of B. subtilis (91), two pairs of prolines facing each other in the transmembrane region were proposed to be crucial for signal sensing and transduction (92). We note that in the TM domain of YukC, two partially conserved prolines, P^231^ and P^245^ (Fig. S5C), are positioned in the dimer in a manner strikingly similar to that of those found in the model of DesK. We demonstrate that mutation of YukC’s proline 231, located at the center of the transmembrane region, partially affected B. subtilis’s ability to outcompete recipient cells (Fig. 4C) without affecting YukC’s stability (Fig. S5D). The decreased fitness of the P231A strain would be caused by a change in the signaling properties of YukC’s stalk due to the absence of this proline. Interestingly, a proline in the transmembrane region of the ATPase EccC_5_ was recently proposed to promote flexibility in this region, influencing the formation of the T7SSa central pore (34). It will be interesting to investigate whether and how mutations in this and other transmembrane proline residues affect the YukC-YueB interaction, since YueB has been proposed to form a central pore together with the ATPase (10).

### Conclusion.

Altogether, our results suggest that pseudokinases have three key roles in the T7SSb secretion mechanism: substrate recruitment (LXG and WXG effectors), energy coupling (based on the cytoplasmic interaction with the ATPase, the dissociation-prone PK dimer, and the flexible β-swap region), and signal transduction (through the transmembrane stalk interacting with the putative pore-forming subunit). Even if more evidence is needed to understand the molecular dynamics behind our proposed model, this work establishes B. subtilis as an important model system for structure-function analyses of T7SSb secretion mechanisms.

## MATERIALS AND METHODS

### Cloning.

The primers used in this work are listed in Table S1. The plasmids are listed in Table S2. Plasmid propagation and construction were performed in E. coli DH5α. Target genes were amplified using Phusion polymerase (Thermo Scientific) and B. subtilis 168 genomic DNA. In the case of pRSFduet/*yueB*.strep, four primers were used to generate annealing overhangs. pJM14 (*amyE*::P*hyperspank*-*gfp*, spectinomycin) was built in a two-way ligation with a PCR-amplified fragment containing the *gfp* gene and an optimized ribosome-binding site (with oligonucleotide primers oJM31 and oJM32 and plasmid pKL147 [93] as the template) and pDR111 (kind gift from D. Z. Rudner) cut with HindIII and NheI (NEB).

10.1128/mbio.00134-22.6 TABLE S1 Primers used in this study. Download Table S1, PDF file, 0.1 MB. Copyright © 2022 Tassinari et al. 2022 Tassinari et al. https://creativecommons.org/licenses/by/4.0/ This content is distributed under the terms of the Creative Commons Attribution 4.0 International license.

### Bacterial strains.

The strains used are listed in Table S3. To engineer B. subtilis T7SSb mutant strains, we initially purchased strains from the Bacillus Genetic Stock Center (BGSC) that present mutations *yukE*::*erm*, *yukD*::*erm*, *yukC*::*erm*, *yukB*::*erm*, *yueB*::*erm*, and *yueC*::*erm* in the B. subtilis 168 *trpC*^−^ genetic background (BKE31910 to BKE31850), in which the erythromycin (Erm) resistance cassettes are flanked by *lox* sites. The respective markerless deletions used for our experiments were obtained by eliminating the *erm* cassette from each B. subtilis mutant. For this purpose, each strain was transformed with the thermosensitive vector pDR244 encoding the Cre recombinase (94). pDR244 was eliminated by growing the cells at 42°C on LB agar plates, and mutants were tested by colony PCR.

10.1128/mbio.00134-22.8 TABLE S3 Strains used in this study. Download Table S3, PDF file, 0.1 MB. Copyright © 2022 Tassinari et al. 2022 Tassinari et al. https://creativecommons.org/licenses/by/4.0/ This content is distributed under the terms of the Creative Commons Attribution 4.0 International license.

For bacterial competition assays, the WT B. subtilis strain 168 CA or markerless strains with T7SSb subunits deleted were used as attackers. The B. subtilis 168 CA strain, which lacks the effector-immunity protein pair YxiD-YxxD (B. subtilis Δ*yxiD-yxxD*), was used as recipient. This strain was made by transforming B. subtilis 168 CA with the pJM14 integrative vector carrying *yxiD-yxxD* flanking sites inserted using the Gibson assembly kit (NEB). To analyze the *in vivo* effects of YukC mutations P231A and H209C on bacterial competition, we decided to complement the Δ*yukC* strain using either the wild-type or mutant *yukC* gene. In our hands, *yuk* operon deletions were not complemented by genes inserted on replicative plasmids (or inducible or constitutive plasmids). We planned to reinsert the *yukC* gene at its original location in the *yuk* operon by using homologous recombination in the B. subtilis 168 *yukC*::*erm* strain (primers in Table S1). In the pETduet plasmid, which is unable to replicate in B. subtilis, we inserted the *yukE-yukD-yukC*-*yukB* fragment (500 bp) by Gibson assembly. In this initial plasmid (FG01), we inserted a kanamycin cassette flanked by *lox* sites. This lox-Kan-lox cassette was amplified using pDR110 as a template and inserted upstream from *yukC* by Gibson assembly, creating the plasmid FG02. The kanamycin cassette was subsequently removed using pDR244 (as explained above), thus obtaining the markerless strains that were used as attackers in the bacterial competition experiments whose results are shown in Fig. 4C.

### B. subtilis genome extraction.

A fresh single colony was inoculated into 2 mL of LB medium and grown until mid- to late exponential phase (λ = *A*_600_ of ~0.8). Cells were pelleted and resuspended in 200 μL of lysis buffer (20 mM Tris-HCl, pH 7.5, 2 mM EDTA, 20 mg/mL lysozyme, 1.2% Triton X-100). The bacterial solution was incubated for 30 min at 37°C, and DNA was extracted using the DNeasy blood and tissue kit (catalog number 69504; Qiagen).

### Transformation of B. subtilis.

An overnight B. subtilis culture grown from a single colony in LB was diluted 1:100 in 5 mL of SpI medium [2 g/L (NH4)_2_SO_4_, 14 g/L K_2_HPO_4_, 6 g/L KH_2_PO_4_, 1 g/L Na-citrate·2H_2_O, 0.2 g/L MgSO_4_·7H_2_O, 0.5% glucose, 0.1% yeast extract, 0.02% Casamino Acids] and grow at 37°C with shaking at 180 rpm. An amount of 0.5 mL of late-exponential-phase cells was added to 4.5 mL of SpII medium (SpI supplemented with 0.5 mM CaCl_2_ and 2.5 mM MgCl_2_) and grown for 90 min at 37°C with shaking at 100 rpm. The culture was supplemented with 1 mM EGTA and incubated for an additional 10 min. One to 10 μg of purified DNA was added to 300 μL of B. subtilis cells and incubated at 30°C for 90 min with 100-rpm shaking. The culture was selected on LB solid medium supplemented with 2 μg/mL Erm.

### Bacterial competition assay.

Single colonies from glycerol stocks (Table S3) were grown on LB solid medium overnight at 30°C before being inoculated into 3 mL of liquid LB supplemented with 100 μg/mL spectinomycin (Spec) for B. subtilis recipient cells. Cells were diluted 1:100 in 4 mL of secretion induction medium (SIM) (M9 medium supplemented with 10% LB, 2.4% glycerol, 0.4% glucose, 10 μg/mL thiamine, 75 μg/mL Casamino Acids, 1 mM MgSO_4_, 0.1 mM CaCl_2_, 50 μM FeCl_3_, and 100 μM citrate) without antibiotics. The B. subtilis recipient strain was induced by the addition of 200 μM IPTG (isopropyl β-d-1-thiogalactopyranoside) at an *A*_600_ of 0.2. Two milliliters of each bacterial culture at an *A*_600_ of 0.8 was pelleted by centrifugation at 8,000 × *g* for 5 min and resuspended in SIM to an *A*_600_ of 10. Attacker and recipient cells were mixed in a 5:1 ratio and adjusted to 100 μL with SIM. Amounts of 12.5 μL of each bacterial mixture were spotted in duplicate on solid SIM supplemented with 200 μM IPTG to promote GFP expression in the recipient, and the plates were incubated overnight at 30°C. To visualize the GFP fluorescence, the competition plates were imaged in a Bio-Rad ChemiDoc system, using the Alexa Fluor 488 program. Bacteria from each spot were resuspended in 1 mL of LB liquid medium and then diluted 10^−3^ to 10^−6^ times. Amounts of 100 μL of the dilutions were plated on LB solid medium supplemented with Spec at 100 μg/mL. For each competition experiment, six spots were analyzed from 3 independent experiments. After overnight incubation at 37°C, the surviving B. subtilis recipient cells were counted to calculate the numbers of CFU of survivors.

### Live microscopy.

Amounts of 2 μL of each B. subtilis competition mixture (5:1 ratio) were spotted onto pads made of SIM supplemented with 2% agarose. Live imaging was performed using a Zeiss Axio Observer Z1 microscope fitted with an Orca Flash 4 V2 scientific complementary metal oxide semiconductor (sCMOS) camera (Hamamatsu) and a Plan-Apo 63×/1.4 Ph3 oil objective (Zeiss) and analyzed using Fiji software (95). For B. subtilis 168 or B. subtilis Δ*yukC* versus B. subtilis Δ*yxiD-yxxD*, frames were collected every 20 min during overnight growth at 30°C, using phase-contrast and epifluorescence microscopy (585 nm with a 40-ms exposure time).

### SPP1 infection assay.

A fresh B. subtilis colony was inoculated into 3 mL of liquid LB and grown overnight at 37°C with shaking at 180 rpm. The culture was diluted 1:100 in 3 mL of LB, and once it approached the exponential phase (*A*_600_ of 0.5), 250 μL was mixed with 5 mL of LB top agar (0.6% agar) supplemented with 10 mM CaCl_2_. The mixture was quickly poured onto solid LB and solidified. Amounts of 10 μL of serially diluted SPP1 were spotted onto the top agar surface, and the plate was incubated at 37°C overnight. The SPP1 stock solution was kindly provided by P. Tavares.

### BACTH assay.

The bacterial two-hybrid (BACTH) assay was performed according to the manufacturer’s instructions (Euromedex). The plasmids for the BACTH assays are listed in Table S2. Briefly, 50 μL of E. coli BTH101 cells (68) were cotransformed with 20 ng of pKT25/*X* or pKNT25/*X* and 20 ng of pUT18/*X* or pUT18C/*X*, where *X* indicates the gene of interest, and selected on solid LB medium with ampicillin (Amp; 100 μg/mL) and kanamycin (Kan; 50 μg/mL). As a negative control, empty pKT25 and PUT18C vectors were used, while the vectors carrying the *zip* gene were used as a positive control. Selected colonies were grown in 5 mL of LB supplemented with the selective antibiotics until reaching an *A*_600_ of 0.4, and 1 μL was then spotted on solid LB medium supplemented with 40 μg/mL of X-Gal (5-bromo-4-chloro-3-indolyl-β-d-galactoside; freshly prepared in dimethylformamide) (Euromedex), Amp and Kan (100 and 50 μg/mL, respectively), and 0.5 mM IPTG. As an alternative to Amp, carbenicillin (Carb) at 50 μg/mL was used because of its higher stability. To increase the number of colonies tested, a variation of this protocol was developed. Briefly, after double transformation, BTH101 cells were resuspended in 2 μL of LB. Then, 1 μL of this mixture was directly spotted onto solid LB medium supplemented with Carb at 50 μg/mL, Kan at 50 μg/mL, 5 mM IPTG, and 40 μg/mL X-Gal. Each transformation was performed at least twice, and 3 to 6 colonies per transformation were tested for each interaction. Therefore, each BACTH assay reflects the results for a minimum of 6 different colonies. The plates were incubated at 25°C and imaged after 48 h. The *yukC* constructs used for the BACTH assays were full length unless otherwise indicated.

### Copurification of YukC (full length or PK) with YukB_Δ256_ (FHA domains).

Two liters of LB medium supplemented with Carb at 50 μg/mL and Spec at 50 μg/mL was inoculated with E. coli BL21(DE3) (96) cotransformed with pCDF/*yukB*_Δ256B_.his and either pRSF/*yukC*_FL_.strep for full-length YukC copurification or pRSF/*yukC*_Δ217_.strep for YukC-PK. In both cases, the buffer solutions were supplemented with 0.5 mM TCEP [Tris(2-carboxyethyl)phosphine hydrochloride]. When pRSF/*yukC*_FL_.strep was used, the membrane fraction was isolated by ultracentrifugation and detergent solubilized as described below in “Copurification of YukC and YukE.” For the affinity chromatography step, samples were first loaded into 5-mL StrepTrap columns (GE Healthcare) and washed with 50 mL of purification buffer (25 mM HEPES, pH 8, 175 mM NaCl, 0.03% Triton X-100), and then proteins were eluted directly into a 1-mL HisTrap column (GE Healthcare) using purification buffer supplemented with 2.5 mM desthiobiotin (IBA). The HisTrap column was then washed with 20 mL of purification buffer supplemented with 40 mM imidazole, and the proteins were eluted with the same buffer supplemented with 0.5 M imidazole. The eluted proteins were separated by SDS-PAGE and detected by using TGX stain-free gels and immunoblotting.

### YukC_FL_-YueB copurification.

Six liters of terrific broth (TB) supplemented with Carb at 50 μg/mL, Kan at 50 μg/mL, and 0.8% glycerol was inoculated with E. coli C43(DE3) (97) cotransformed with pET15b/*yukC*_FL_.his and pRSF/*yueB*.strep. YukC_FL_ and YueB were induced as described for purification of the YukC_413_.strep construct (see below). Cells were resuspended in 200 mL of cold lysis buffer (50 mM HEPES, pH 8, 300 mM NaCl, 1 mM EDTA, 5% glycerol, 4 tablets of protease inhibitors; Roche). Cells were lysed, and the membrane fraction collected as described for the purification of the YukC_413_.strep construct. Membranes were resuspended in 20 mL of cold solubilization buffer (25 mM HEPES, pH 8, 300 mM NaCl, 2.5% glycerol, 1 mM TCEP, 1 tablet of protease inhibitors). Membrane solubilization was achieved by adding 0.5% LDAO (lauryldimethylamine-*N*-oxide) and then incubating for 40 min under moderate shaking at 4°C. For the affinity chromatography, the sample was first loaded into a 5-mL StrepTrap column (GE Healthcare) and washed with 50 mL of purification buffer (25 mM HEPES, pH 8, 175 mM NaCl, 0.02% LDAO). The StrepTrap column was washed with purification buffer containing 0.004% lauryl maltose neopentyl glycol (LMNG; Anatrace). Proteins were eluted directly into a 1-mL HisTrap column (GE Healthcare) using purification buffer supplemented with 2.5 mM desthiobiotin (IBA). The HisTrap column was then washed and eluted as described for YukC-YukB_Δ256_ (FHA) copurifications. The eluted proteins were separated by SDS-PAGE and detected by Coomassie staining and immunoblotting in parallel.

### Copurification of YukC and YukE.

Two liters of LB supplemented with 50 μg/mL Carb and 50 μg/mL Kan was inoculated with E. coli BL21(DE3) cotransformed with pRSF/*yukC*_413_.strep and pET15b/*yukE*.his. YukC and YukE were induced, and cells grown as described below for the YukC_413_.strep construct. The YukC-YukE complex was isolated from the membrane fraction by applying the protocol used for the purification of the YukC_413_.strep construct, described below, except for the presence of 1% (vol/vol) Triton X-100 instead of 1% Cymal-6 (6-cyclohexyl-1-hexyl-β-d-maltoside; Anatrace). Affinity chromatography was performed as described for YukC-YukB_Δ256_ (FHA) copurifications. The elution fractions were analyzed by Coomassie-stained SDS-PAGE gels. The YukC and YukE identities were confirmed by LC-MS/MS.

### Immunoblots.

Proteins separated on SDS-PAGE were transferred onto a polyvinylidene difluoride (PVDF) membrane (0.2 μM, Immobilon; Millipore) previously activated by ethanol and preequilibrated with transfer buffer (25 mM Tris, 192 mM glycine, 0.1% SDS, 20% ethanol) using a Trans-Blot Turbo system (Bio-Rad). The PVDF membrane was blocked for 1 h in blocking solution (PBS, 0.1% Tween 80 from Sigma-Aldrich, 4% milk powder). The primary antibodies (anti-Strep antibody from IBA diluted 1:1,000 and anti-His_6_ antibody from Sigma-Aldrich diluted 1:2,000) were incubated overnight at 4°C under gentle shaking. The PVDF membrane was washed 3 times for 10 min in PBS-Tween. The horseradish peroxidase (HRP)-conjugated anti-mouse secondary antibody (Sigma-Aldrich) was diluted 1:5,000 in PBS-Tween and incubated for 1 h. The PDVF membrane was washed 3 times for 10 min in PBS-Tween. The blots were developed using an ECL kit (Amersham Biosystems) in a ChemiDoc system (Bio-Rad).

### YukC_413_ purification for crystallization.

A single colony of E. coli BL21(DE3) transformed with pRSF/*yukC*_413_.strep was inoculated into 100 mL of LB supplemented with Kan 50 μg/mL. After overnight growth at 37°C with shaking at 180 rpm, the preculture was diluted 1:50 in 4 L of LB medium supplemented with Kan (50 μg/mL) and incubated at 37°C with shaking at 180 rpm to an *A*_600_ of 0.8. Expression was induced by the addition of 0.5 mM IPTG, and the temperature was decreased to 16°C overnight. Cells were collected by centrifugation at 6,000 × *g* for 20 min at 4°C. During purification, the sample was kept on ice unless otherwise specified. Cells were resuspended in 100 mL of cold lysis buffer (50 mM HEPES, pH 8, 175 mM NaCl, 5% glycerol, 1 mM EDTA, and 2 tablets of protease inhibitors; Roche) and then incubated at room temperature (RT) for 20 min under moderate shaking in the presence of 0.5 mg/mL lysozyme (Sigma-Aldrich) and for an additional 10 min after the addition of 0.1 mg/mL DNase, 5 mM MgCl_2_. Cell lysis was obtained in 3 cycles of an Emulsiflex homogenizer (Avestin, USA) operating at about 20,000 lb/in^2^. Unbroken cells were centrifuged for 15 min at 12,000 × *g*. Membranes were collected using a Ti45 rotor (Beckman Coulter) running at 100,000 × *g* for 1 h (at 4°C) and resuspended in 20 mL of cold solubilization buffer (25 mM HEPES, pH 8, 175 mM NaCl, 2.5% glycerol, 1 tablet of protease inhibitors). Twenty milliliters of 2% Cymal-6 (Anatrace) solution was added slowly to the membrane suspension, and the mixture incubated at RT for 30 min under moderate shaking. Unsolubilized material was removed by ultracentrifugation using the SW32 rotor (Beckmann) at 100,000 × *g* for 45 min. The cleared solubilized material was loaded at 0.8 mL/min on a preequilibrated 5-mL StrepTrap column (GE Healthcare) mounted on an Akta purifier (GE health care) preequilibrated with purification buffer (25 mM HEPES, pH 8, 175 mM NaCl, 0.04% Cymal-6). The affinity column was washed with 50 mL of purification buffer, and YukC was eluted using the same buffer supplemented with 2.5 mM desthiobiotin (Sigma). The eluted fractions and the intermediate purification steps were evaluated by SDS-PAGE. Finally, 5 mL of purified YukC was injected onto a Superdex 200-pg 16/600 column to further eliminate contaminant proteins. The yield of protein was approximately 5 mg/L bacterial culture.

### YukC crystallization and structure determination.

Crystals of the YukC_413_ construct were obtained by hanging-drop vapor diffusion in 0.4 μL with a 1:1 ratio between the protein solution (20 mg/mL YukC_413_, 25 mM HEPES, pH 8, 175 mM NaCl, 0.04% Cymal-6) and the crystallization solution (0.1 M MgCl_2_, 50 mM HEPES, pH 7.5, 15% [wt/vol] polyethylene glycol [PEG 2000]). Two-hundred-nanoliter drops were dispensed by a mosquito crystal robot (TTP Labtech) at 4°C, and crystals appeared within 3 days. The crystals were frozen in liquid nitrogen with a 50% Paratone N (Hampton Research), 50% paraffin oil solution as the cryoprotectant. A 2.6-Å-resolution data set was collected on beamline ID30A-3 at the European Synchrotron Radiation Facility (ESRF) in Grenoble, France. Data were processed with autoPROC (98), and the structure (PDB ID 6Z0F) was solved by a two-step molecular replacement approach with PHASER (99). RMSDs were calculated with MolProbity (100) within the PHENIX crystallographic software suite.

A first molecular-replacement search was performed using the coordinates of the dimeric extracellular portion of G. thermodenitrificans EssB (PDB ID 2YNQ) as the probe, followed by a new search using the coordinates of the pseudokinase domain from the same protein (PDB ID 4ANO). Once a solution including both the intracellular and extracellular domains was identified, the missing part of the molecule, including the transmembrane domain, was modeled by combining automated tracing with Phenix AutoBuild (101) and manual rebuilding with Coot (102). The overall model was then subjected to alternating cycles of inspection with Coot and refinement with BUSTER (103).

### Analysis of the YukC dimer interface.

The coordinates of YukC were analyzed with the PISA server (http://www.ebi.ac.uk/msd-srv/prot_int/cgi-bin/piserver) (104) to evaluate dimerization surfaces (*A*^2^) for different regions of YukC, as well as their corresponding Δ*^i^G* values, and to identify residues involved in H bonds or salt bridges.

### Analysis of the β-swap domain.

**(i) Cysteine substitution construction.** Codon substitutions were introduced into the pBE-S/*yukC.*his vector by site-directed mutagenesis using complementary pairs of oligonucleotides bearing the desired substitution (Table S1) (synthesized by Sigma-Aldrich) and *Pfu* Turbo DNA polymerase (Agilent Technologies). All constructs were verified by DNA sequencing (Eurofins Genomics).

### (ii) Cysteine cross-linking assay.

Overnight cultures of B. subtilis cells producing the wild-type 6×His-tagged YukC or its cysteine derivatives were diluted to an *A*_600_ of 0.1 in LB. Once the cultures reached an *A*_600_ of 0.8, *N*-ethyl maleimide (Sigma-Aldrich) was added at a final concentration of 5 mM to block all free thiol groups. After 20 min of incubation at 37°C, cells were harvested, resuspended in lysis buffer (20 mM Tris-HCl, pH 7.5, 10 mM EDTA, 10 mM MgCl_2_, 1 mg/mL lysozyme, 100 μg/mL DNase) supplemented with protease inhibitors (Roche), frozen for 15 min at −80°C, thawed at 37°C before the addition of Laemmli loading buffer supplemented or not with 8% β-mercaptoethanol (β-me), and boiled for 5 min. Total extracts were run on 10% acrylamide SDS-PAGE gels and transferred onto nitrocellulose. YukC and YukC multimers were detected with histidine-specific antibody (clone AD1.1.10; Bio-Rad).

### Comparison of YukC structure.

Structure similarity for YukC was searched using the web tool DALI (71). The selected protein sequences were aligned with the web tool T-COFFEE (105), and the structures were superimposed with the software UCSF Chimera (106).

### Nanoscale differential scanning fluorimetry (nanoDSF).

Thermal denaturation was performed on the Prometheus NT.48 (NanoTemper) from 20 to 95°C with 80% excitation laser power and a heating rate of 2°C/min. The tryptophan fluorescence emissions were monitored at 330 nm and 350 nm as a function of increasing temperature. The purified YukC_413_.strep construct and PknB were diluted to a concentration of 1 mg/mL in the presence or in the absence of 1 mM ATP in 25 mM HEPES, pH 8, 175 mM NaCl, 2 mM MgCl_2_, 0.04% Cymal-6. The sample was filled into the capillaries, and the emissions at 350 nm and 330 nm were measured. The intrinsic fluorescence signal expressed by the 350-nm/330-nm emission ratio was plotted as a function of temperature. Three replicates were performed for each protein. The average values of the replicates were calculated and are plotted in Fig. S4C.

### Analyses of YukC conservation and stability.

The conservation of YukC was evaluated using HMMER software (http://hmmer.org). The sequence logo reported in Fig. S5C is based on this alignment, and it was created at https://weblogo.berkeley.edu/logo.cgi.

Analysis of the stability of YukC.His^P231A^ was performed by expressing wild-type YukC (YukC.His) and the mutant (YukC.His^P231A^) in E. coli, together with the membrane protein TseB as a control. Cell samples were collected at different time points after protein synthesis was blocked by chloramphenicol and were analyzed by Western blotting using antibodies directed against the His tag (for YukC) or against TseB. The fluorescence signals of YukC.His and YukC.His^P231A^ at each time point were normalized by the corresponding ones of TseB, and their decay rates were compared.

### Data availability.

The structure of YukC/EccB dimer is available in the Protein Data Bank database with the accession number 6Z0F.
