# Supplementary figures and images for: The Antibacterial Type VII Secretion System of Bacillus subtilis: Structure and Interactions of the Pseudokinase YukC/EssB
Source: mBio. 2022 Sep 26;13(5):e00134-22. doi: 10.1128/mbio.00134-22 (PMC9600267; doi:10.1128/mbio.00134-22)

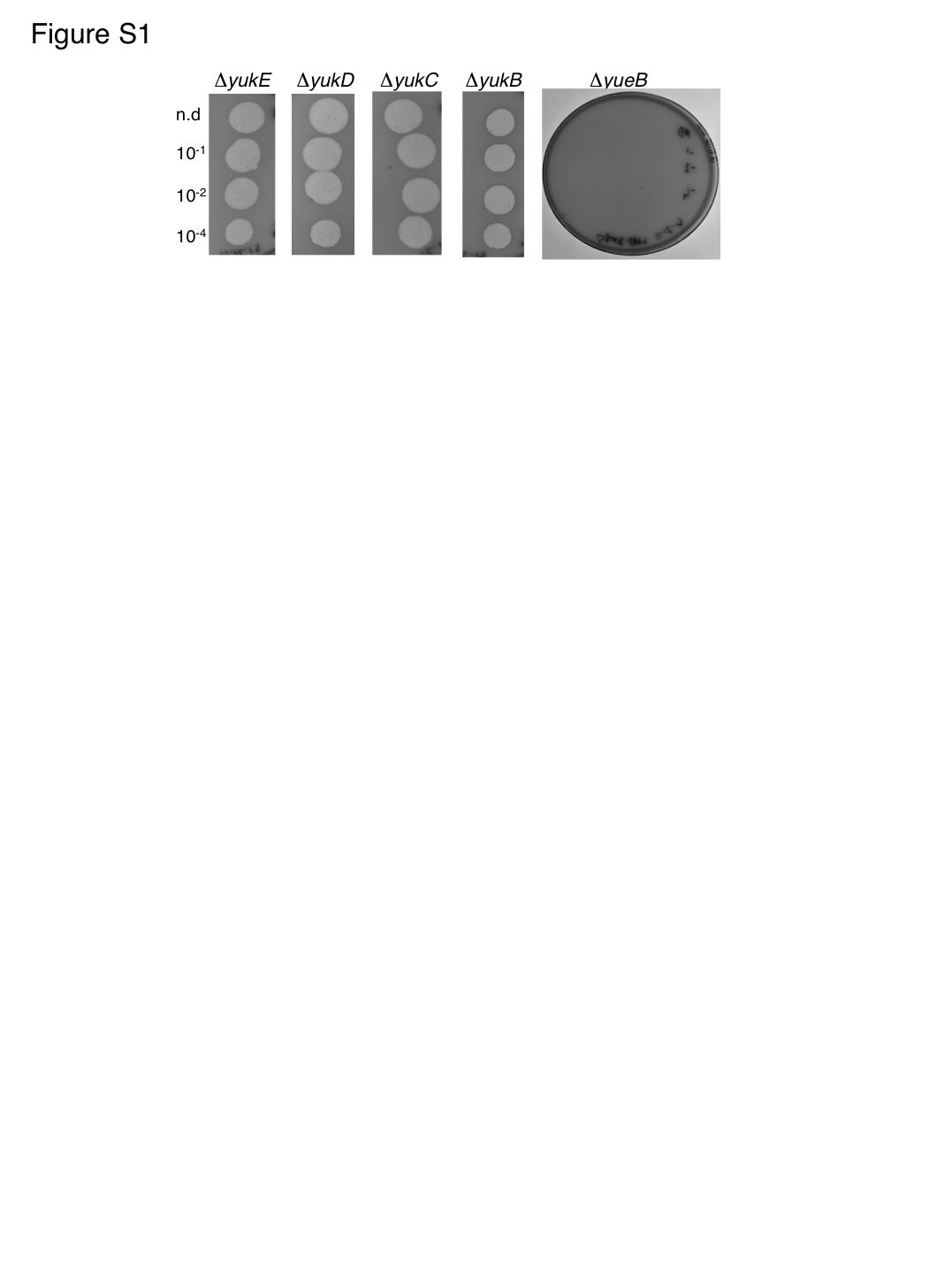

Supplement: FIG S1 [file mbio.00134-22-s0006.jpg]

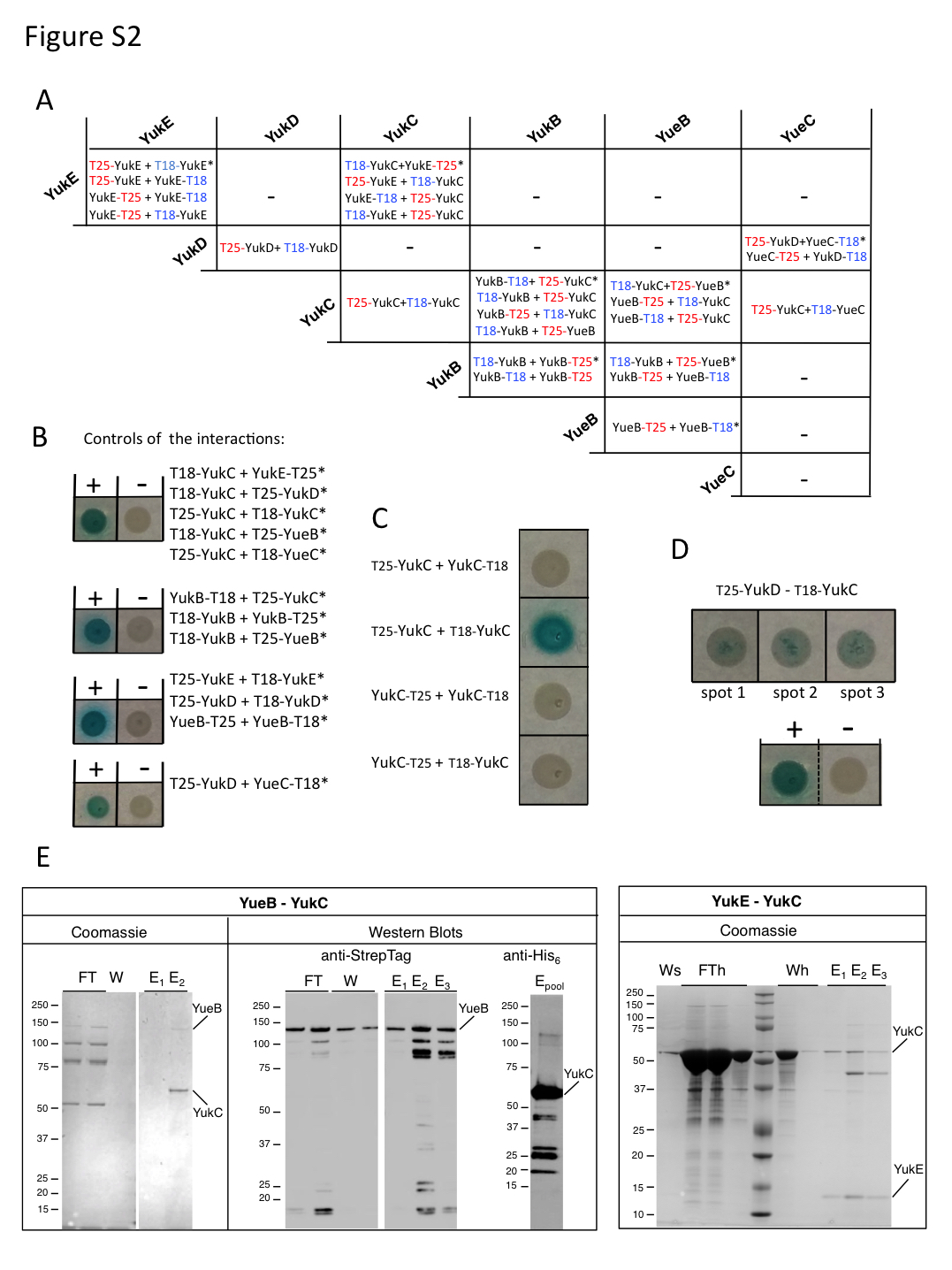

Supplement: FIG S2 [file mbio.00134-22-s0007.jpg]

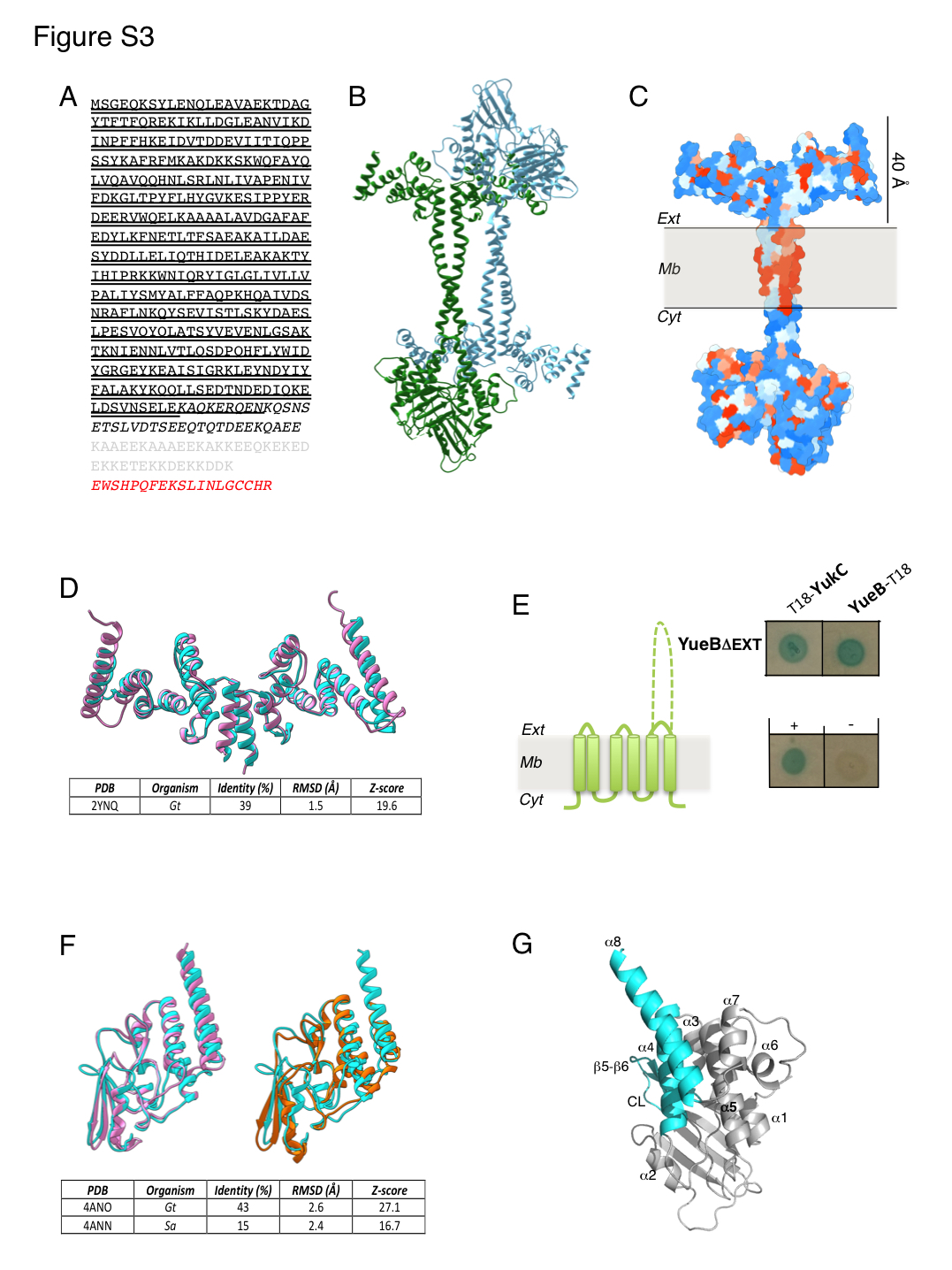

Supplement: FIG S3 [file mbio.00134-22-s0008.jpg]

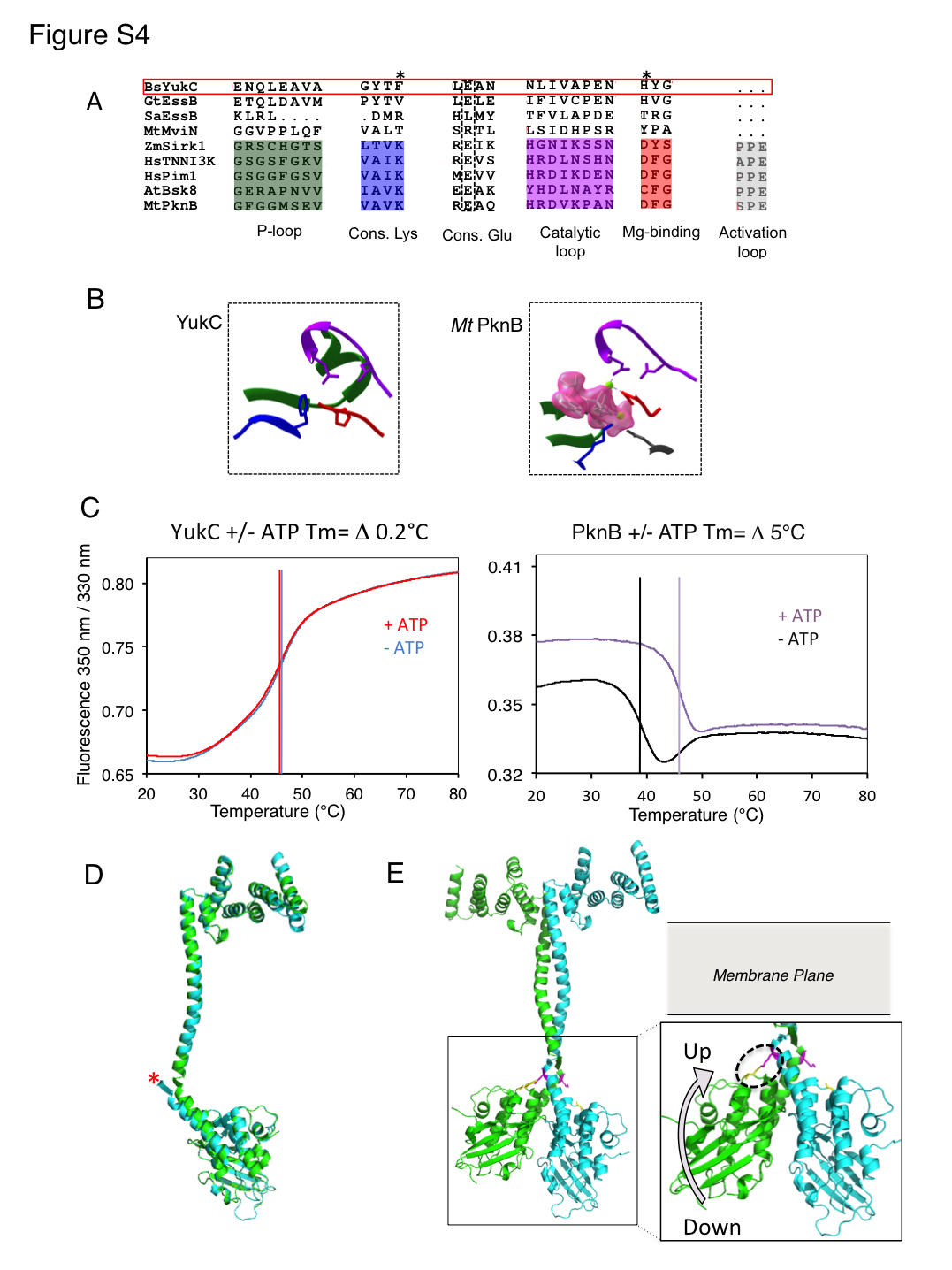

Supplement: FIG S4 [file mbio.00134-22-s0009.jpg]

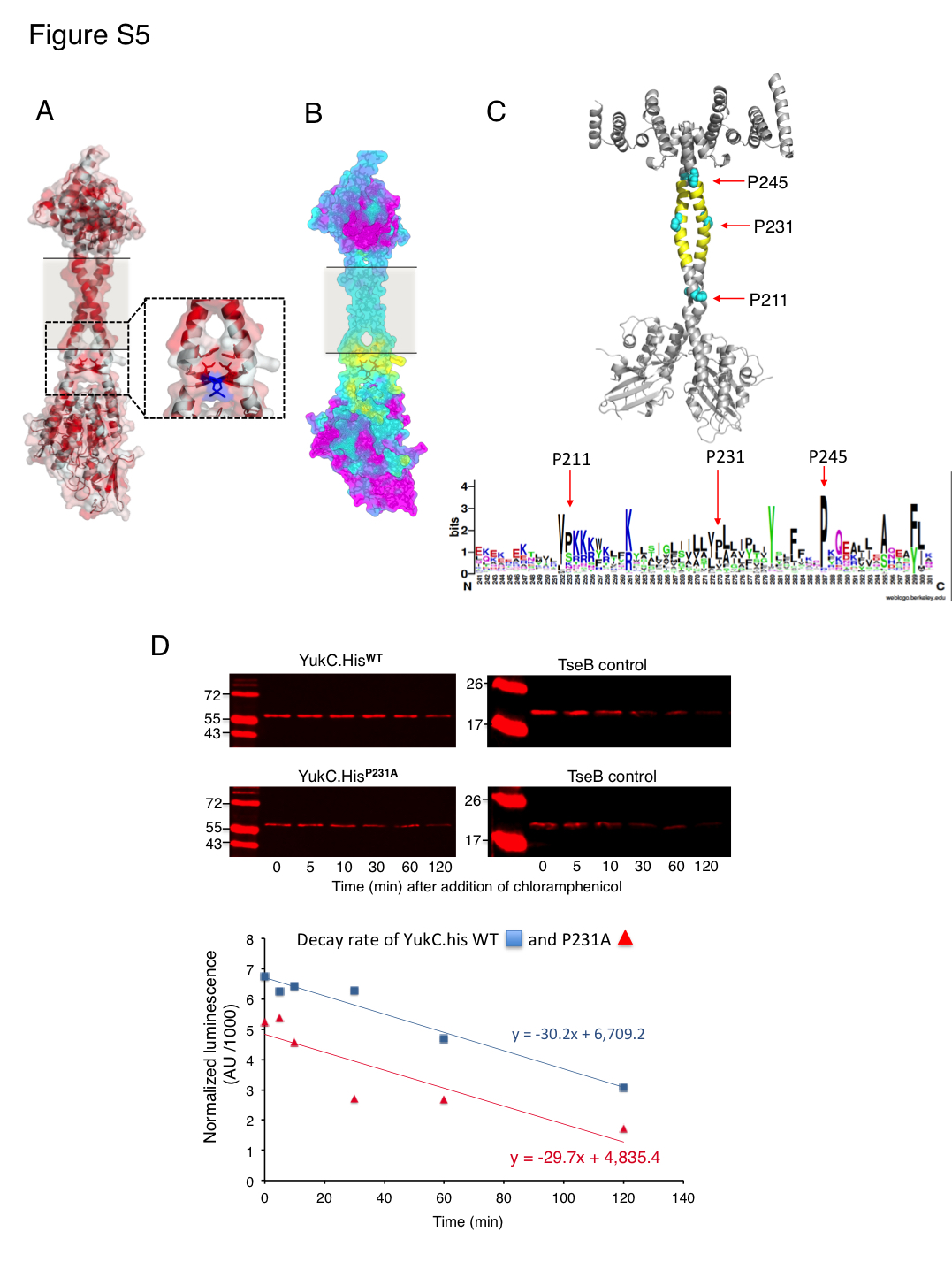

Supplement: FIG S5 [file mbio.00134-22-s0010.jpg]
