# Supplementary material for: The Antibacterial Type VII Secretion System of Bacillus subtilis: Structure and Interactions of the Pseudokinase YukC/EssB
Source: mBio. 2022 Sep 26;13(5):e00134-22. doi: 10.1128/mbio.00134-22 (PMC9600267; doi:10.1128/mbio.00134-22)
Supplement: TABLE S2 [file mbio.00134-22-s0004.pdf]

Table S2.

Plasmid	Host - Resistance	Reference
**BACTH assay**		
pKT25/*yukE*	*E. coli*- Kan - 50 µg/mL	This study
pKNT25/*yukE*	*E. coli*- Kan - 50 µg/mL	This study
pKT25/*yukD*	*E. coli*- Kan - 50 µg/mL	This study
pKNT25/*yukD*	*E. coli*- Kan - 50 µg/mL	This study
pKT25/*yukC*	*E. coli*- Kan - 50 µg/mL	This study
pKNT25/*yukC*	*E. coli*- Kan - 50 µg/mL	This study
pKNT25/*yukB*	*E. coli*- Kan - 50 µg/mL	This study
pKT25/*yukB*Δ256	*E. coli*- Kan - 50 µg/mL	This study
pKT25/*yueB*	*E. coli*- Kan - 50 µg/mL	This study
pKNT25/*yueB*	*E. coli*- Kan - 50 µg/mL	This study
pKT25/*yueB*Δ31-817	*E. coli*- Kan - 50 µg/mL	This study
pKNT25/*yueB*Δ31-817	*E. coli*- Kan - 50 µg/mL	This study
**Protein expression**		
pKT25/*yueC*	*E. coli*- Kan - 50 µg/mL	This study
pKNT25/*yueC*	*E. coli*- Kan - 50 µg/mL	This study
pKNT25/$yxiD_N$	*E. coli*- Kan - 50 µg/mL	This study
pUT18/*yukE*	*E. coli* -Amp 100 µg/mL	This study
pUT18C/*yukE*	*E. coli* -Amp 100 µg/mL	This study
pUT18/*yukD*	*E. coli* -Amp 100 µg/mL	This study
pUT18C/*yukD*	*E. coli* -Amp 100 µg/mL	This study
pUT18/*yukC*	*E. coli* -Amp 100 µg/mL	This study
pUT18C/*yukC*	*E. coli* -Amp 100 µg/mL	This study
pUT18C/*yukB*	*E. coli* -Amp 100 µg/mL	This study
pUT18/*yukB*	*E. coli* -Amp 100 µg/mL	This study
pUT18/*yukB*Δ256	*E. coli* -Amp 100 µg/mL	This study
pUT18/*yueB*	*E. coli* -Amp 100 µg/mL	This study

Table S2 (continues)

pUT18/*yueC*	*E. coli* -Amp 100 µg/mL	This study
pUT18C/*yueC*	*E. coli* -Amp 100 µg/mL	This study
pUT18/*yxiD*$_N$	*E. coli* -Amp 100 µg/mL	This study
pUT18C/*yxiD*$_N$	*E. coli* -Amp 100 µg/mL	This study
pBES_*yukC*.his	*E. coli* -Amp 100 µg/mL<br>*B. subtilis*- Kan 30 µg/mL	This study
pET15b_*yukC*.his	*E. coli* -Amp 100 µg/mL	This study
pRSFduet_*yukC*.strep	*E. coli*- Kan - 50 µg/mL	This study
pRSFduet_*yukC*Δ413strep	*E. coli*- Kan - 50 µg/mL	This study
pET15b_*yukE*.his	*E. coli* -Amp 100 µg/mL	This study
pCDF_*yukB*Δ256.his	*E. coli*- Spec - 50 µg/mL	This study
pCDFduet_*yukB*.HA	*E. coli*- Spec - 50 µg/mL	This study
pRSFduet_*yueB*.strp	*E. coli*- Kan - 50 µg/mL	This study
**Competition assays**		
pDR244	*E. coli* -Amp 100 µg/mL<br>*B. subtilis*- Spec 100 µg/mL	(63)
pJM14_Δ*yxiD-yxxD*	*E. coli* -Amp 100 µg/mL<br>*B. subtilis*- Spec 100 µg/mL	This study
**YukC complementation**		
FG01	*E. coli* -Amp 100 µg/mL	This study
FG02	*E. coli* -Amp 100 µg/mL	This study

Table S2. Plasmids used in this study
