# Supplementary material for: The Antibacterial Type VII Secretion System of Bacillus subtilis: Structure and Interactions of the Pseudokinase YukC/EssB
Source: mBio. 2022 Sep 26;13(5):e00134-22. doi: 10.1128/mbio.00134-22 (PMC9600267; doi:10.1128/mbio.00134-22)
Supplement: TABLE S1 [file mbio.00134-22-s0003.pdf]

Table S1.

Primers	Sequence
**MluI _yukChis**	TTAACGCGTATGTCAGGTGAACAAAAATCATATTTAG
**yukChis_XbaI**	AATCTAGATTTGTCATCTTTTTCTCATCTTTC
**NdeI _yukChis**	GGGCATATGTCAGGTGAACAAAAATCATATTTAGAAAACC
**yukChis_NotI**	GATGGCGGCCGCTTTGTCATCTTTTTCTCATCTTTCTTTTCTG
**NdeI_yukCstrep -**	GGGCATATGTCAGGTGAACAAAAATCATATTTAGAAAACC
**yukCstrep_KpnI**	CCCGGTACCTTTGTCATCTTTTTCTCATCTTTCTTTTCTG
**pRSFduet_yukCΔ413strep**	TTCTGCTTGTTTTTCTTCGTCTGTTTGTGTCTG
**pRSFduet_yukCΔ413strep**	GAGTGGAGCCACCCGCAGTTC
**NdeI _yukE.his**	GGCATATGGCAGGATTAATTCGTGTCACACCCGAAG
**yukE.his_ BamHI**	GGGGATCCTTATCCGCGGATTTGATTTGCGATGTCTTGGTC
**pCDFduet_yukBΔ256.his**	GTGATGATGCCCTCTGTTGGTTTGATCGCTC
**pCDFduet_yukBΔ256.his**	CATCACCACTAATCGAGTCTGGTAAATACCCATACGATGTTCC
**Nde_yukB.HA**	GGGCATATGCTGCCATTCGGCCGTAAGG
**yukB.HA_XhoI**	GGGCTCGAGAATCTAGCACTCTCCCTTTCAGCAGAAC
**NdeI_yueB.strep**	TATGATGACAGAACAACGAAAAAGCTTGATCAAG
**yueB.strep_KpnI**	GTACCCGCTTCATACGTTTCATCGCTTTC
**NdeI_yueB.strep**	TGATGACAGAACAACGAAAAAGC
**YueB.strep_KpnI**	CCGCTTCATACGTTTCATCGCTTTC
**YukC-Y207C**	GAAGCCAAAGCAAAAACA*TGC*ATACATATCCCGAGAAAG
	CTTTCTCGGGATATGTAT*GCA*TGTTTTTGCTTTGGCTTC
**YukC-I208C**	CCAAAGCAAAAACATAC*TGC*CATATCCCGAGAAAGAAG
	CTTCTTTCTCGGGATATG*GCA*GTATGTTTTTGCTTTGG
**YukC-H209C**	CAAAGCAAAAACATACATA*TGT*ATCCCGAGAAAGAAGTG
	CACTTCTTTCTCGGGAT*ACA*TATGTATGTTTTTGCTTTG
**YukC-I210C**	GCAAAAACATACATACAT*TGC*CCGAGAAAGAAGTGGAAC
	GTTCCACTTCTTTCTCGG*GCA*ATGTATGTATGTTTTTGC
**YukC-R212C**	CATACATACATATCCCG*TGT*AAGAAGTGGAACATCC
	GGATGTTCCACTTCTT*ACA*CGGGATATGTATGTATG
**YukC-I221C**	GGAACATCCAGCGATAT*TGC*GGCCTGGGGCTCATTG
	CAATGAGCCCCAGGCC*GCA*ATATCGCTGGATGTTCC
**YukC-G222C**	CATCCAGCGATATATC*TGC*CTGGGGCTCATTGTTTTG
	CAAAACAATGAGCCCCAG*GCA*GATATATCGCTGGATG
**YukC-L223C**	CCAGCGATATATCGGC*TGC*GGGCTCATTGTTTTGCTTG
	CAAGCAAAACAATGAGCCC*GCA*GCCGATATATCGCTGG
**YukC-P231A**	CATTGTTTTGCTTGTAGCGGCACTTATTTATTCG
	CGA ATA AAT AAG TGC CGC TAC AAG CAA AAC AAT G

**Primers** (continues)	**Sequence**
**FG01 plasmid**	
fragment Fw	ATAGGGGGGCAGGATTAATTCGTGTCACACCCGAAG
fragment Rv	ATTTCGCAGCATCTCTGTACTCGGTTTGACCGTTTCTG
vector Fw	GAGATGCTGCGAAATTTGAACGCCAGCACA
vector Rv	ATCCTGCCCCCCTATAGTGAGTCGTATTAATTTCGCGG
**FG02 plasmid**	
pDR110 Fw	GAAATATTATGATACCGTTCGTATAGCATACATTATACGAAGTTATAAAT
pDR110 Rv	CAGATCCTTTACCGTTCGTATAATGTATGCTATACGAAGTTATCAGC
FG01 Fw	CGAACGGTAAAGGATCTGGTTGAATGTCAGGTGAACA
FG01 Rv	ATACGAACGGTATCATAATATTTCAAGCCGGTCTCCGTTTGT

Table S1. Primers used in this study
