# Supplementary material for: The Antibacterial Type VII Secretion System of Bacillus subtilis: Structure and Interactions of the Pseudokinase YukC/EssB
Source: mBio. 2022 Sep 26;13(5):e00134-22. doi: 10.1128/mbio.00134-22 (PMC9600267; doi:10.1128/mbio.00134-22)
Supplement: TABLE S3 [file mbio.00134-22-s0005.pdf]

Table S3.

Strain	Resistance	Reference
B. subtilis 168	none	(65)
B. subtilis 168 *yukE::erm*	Erytromicin	BGSC
B. subtilis 168 *yukD::erm*	Erytromicin	BGSC
B. subtilis 168 *yukC::erm*	Erytromicin	BGSC
B. subtilis 168 *yukB::erm*	Erytromicin	BGSC
B. subtilis 168 *yueB::erm*	Erytromicin	BGSC
B. subtilis 168 *yueC::erm*	Erytromicin	BGSC
B. subtilis 168 ΔyxiD-yxxD	Spectinomycin	This study
B. subtilis 168 ΔyukE	none	This study
B. subtilis 168 ΔyukD	none	This study
B. subtilis 168 ΔyukC	none	This study
B. subtilis 168 ΔyukB	none	This study
B. subtilis 168 ΔyueB	none	This study
B. subtilis 168 ΔyueC	none	This study
E. coli BL21 (DE3)	none	(101)
E. Coli C43 (DE3)	none	(102)
E. coli BTH101	none	(101)
E. coli DH5α	none	(Invitrogen™)
B. subtilis PseudoWT *(ΔyukC+yukC*)	none	This study
B. subtilis P231A *(ΔyukC+ yukC_P231A)*	none	This study
B. subtilis H209C *(ΔyukC+ yukC_H209C)*	none	This study
BsΔyxxD-yxiD amyE::yxxD	Kanamycin	This study
Bs168 *yxiD::erm*	Erytromicin	BGSC

Table S3. Strains used in this study
